# Source-specific nitrate intake and all-cause mortality in the Danish Diet, Cancer, and Health Study

**DOI:** 10.1007/s10654-024-01133-5

**Published:** 2024-05-28

**Authors:** Nicola P. Bondonno, Pratik Pokharel, Catherine P. Bondonno, Dorit W. Erichsen, Liezhou Zhong, Jörg Schullehner, Kirsten Frederiksen, Cecilie Kyrø, Peter Fjeldstad Hendriksen, Jonathan M. Hodgson, Frederik Dalgaard, Lauren C. Blekkenhorst, Ole Raaschou-Nielsen, Torben Sigsgaard, Christina C. Dahm, Anne Tjønneland, Anja Olsen

**Affiliations:** 1 The Danish Cancer Institute, Copenhagen, Denmark; 2 Nutrition and Health Innovation Research Institute, School of Medical and Health Sciences, Edith Cowan University, Perth, Australia; 3 Medical School, The University of Western Australia, Royal Perth Hospital, Perth, WA Australia; 4 Institute of Agriculture, The University of Western Australia, Royal Perth Hospital, Perth, WA Australia; 5 Department of Groundwater and Quaternary Geology Mapping, Geological Survey of Denmark and Greenland, Aarhus, Denmark; 6 Department of Public Health, Aarhus University, Aarhus, Denmark; 7 Department of Cardiology, Herlev & Gentofte University Hospital, Copenhagen, Denmark; 8 Department of Environmental Science, Aarhus University, Roskilde, Denmark; 9 Danish Big Data Centre for Environment and Health (BERTHA), Aarhus University, Aarhus, Denmark; 10 Department of Public Health, Faculty of Health and Medical Sciences, University of Copenhagen, Copenhagen, Denmark

**Keywords:** Nitrate, Nitrite, Vegetables, Meat, Water, Mortality

## Abstract

**Introduction:**

Nitrate and nitrite are naturally occurring in both plant- and animal-sourced foods, are used as additives in the processing of meat, and are found in water. There is growing evidence that they exhibit a spectrum of health effects, depending on the dietary source. The aim of the study was to examine source-dependent associations between dietary intakes of nitrate/nitrite and both all-cause and cause-specific mortality.

**Methods:**

In 52,247 participants of the Danish Diet, Cancer and Health Study, associations between source-dependent nitrate and nitrite intakes––calculated using comprehensive food composition and national drinking water quality monitoring databases––and all-cause, cardiovascular disease (CVD)-related, and cancer-related mortality over 27 years were examined using restricted cubic splines within Cox proportional hazards models adjusting for demographic, lifestyle, and dietary confounders. Analyses were stratified by factors hypothesised to influence the formation of carcinogenic *N*-nitroso compounds (namely, smoking and dietary intakes of vitamin C, vitamin E, folate, and polyphenols).

**Results:**

Plant-sourced nitrate intake was inversely associated with all-cause mortality [HR_Q5vsQ1_: 0.83 (0.80, 0.87)] while higher risks of all-cause mortality were seen for higher intakes of naturally occurring animal-sourced nitrate [1.09 (1.04, 1.14)], additive permitted meat-sourced nitrate [1.19 (1.14, 1.25)], and tap water-sourced nitrate [1.19 (1.14, 1.25)]. Similar source-dependent associations were seen for nitrite and for CVD-related and cancer-related mortality except that naturally occurring animal-sourced nitrate and tap water-sourced nitrate were not associated with cancer-related mortality and additive permitted meat-sourced nitrate was not associated with CVD-related mortality. No clear patterns emerged in stratified analyses.

**Conclusion:**

Nitrate/nitrite from plant sources are inversely associated while those from naturally occurring animal-sources, additive-permitted meat sources, and tap water-sources are positively associated with mortality.

**Supplementary Information:**

The online version contains supplementary material available at 10.1007/s10654-024-01133-5.

## Introduction

Nitrate and nitrite, often debated dietary components, play multifaceted roles in human health [1]. Nitrate is a naturally occurring compound formed in living and decaying plants and animals [2]. Nitrate (NO^3^_-_) can be reduced to nitrite (NO^2^_-_) by nitrate-reducing bacteria, both endogenously [3] and exogenously [4]. Therefore, nitrate and—albeit at a much lower level—nitrite are found in both plant- and animal-sourced foods. Plants predominantly use nitrate for growth and its concentration varies due to genetic and environmental factors [5]. While nitrate and nitrite occur naturally in animal-sourced foods, they are also permitted food additives, particularly in the context of meat processing [6, 7]. Drinking water contains nitrate from natural soil processes and human activities, with the latter including ammonia-rich fertilizer use, nitrogen-fixing crop cultivation, and wastewater treatment [8]. As such, while the main source of nitrate in the diet is vegetables—particularly green leafy vegetables and root vegetables such as beetroot [9]—we are continually exposed to nitrate and nitrite from a wide variety of sources.

There is growing evidence that dietary nitrate, depending on its source, exhibits a spectrum of health effects [1]. The conversion of vegetable-sourced nitrate to nitric oxide (NO) through the enterosalivary pathway [10] has been demonstrated to reduce blood pressure, improve vascular function, and enhance physical performance in randomised controlled trials [1]. However, under certain conditions, nitrate, after it has been reduced to nitrite, may form carcinogenic *N*-nitroso compounds (NOCs) [11, 12]. Indeed, higher intakes of water- and processed meat-sourced nitrate/nitrite have been linked to a higher risk of several types of cancer [1]. This dichotomy is hypothesised to be governed by factors that influence nitrosation, like the presence of amines and heme iron in meats [13] or antioxidant vitamins and polyphenols in vegetables [14]. Furthermore, the formation of NOCs is hypothesised to be increased in the presence of smoking but inhibited in the presence of vitamin C, vitamin E, folate, and polyphenols [14]. Two studies have reported a lower incidence of cardiovascular disease (CVD)-related mortality in individuals with a high intake of vegetable-sourced nitrate [15, 16]. As other plant sources like fruits, and animal sources have only recently been incorporated into detailed intake databases for large cohort assessments [9, 17], to date no studies have examined associations between plant- and animal-sourced nitrate/nitrite and mortality. There is also a nascent shift in research focus towards distinguishing health outcomes based on naturally occurring versus additive nitrates and nitrites [18, 19]—essential for shaping informed public health policies. With the global health community critically evaluating the consumption of nitrates and nitrites—especially as food additives [20]—and concerns lingering regarding the consumption of high nitrate containing vegetables, it is imperative to differentiate between sources of nitrate and nitrite and whether they are naturally occurring or used as food additives to understand their varied implications on health.

In this light, the present study aimed to examine associations between dietary nitrate and nitrite intake from various sources [all plants, vegetables, animal-based foods where nitrate/nitrite are naturally occurring, meat products where nitrate/nitrite are allowed additives, and drinking water (nitrate only), separately] and all-cause, CVD-related and cancer-related mortality. Secondary aims were to examine consistency of the associations in the presence of factors hypothesised to influence the formation of *NOCs* (namely, smoking and dietary intakes of vitamin C, vitamin E, folate, and polyphenols).

## Methods

### Study population

Between December 1993 and May 1997, the Danish Diet, Cancer, and Health Study recruited 57,053 men and women who were between the ages of 50 and 65 years, who had no history of cancer (excluding non-melanoma skin cancer), and who were living within the greater areas of Copenhagen and Aarhus. Using unique personal identification numbers assigned to all inhabitants of Denmark, the following databases were cross-linked to the cohort on an individual level: The Civil Registration System [21], The National Death Registry [22], The Danish National Patient Register (DNPR) [23], The Danish National Prescription Registry [24], the Register for Selected Chronic Diseases (RUKS), and the Education Registry [25]. We excluded participants with prevalent CVD (*n* = 2705), defined as record of ischemic heart disease, ischemic stroke, peripheral artery disease, atrial fibrillation or heart failure prior to baseline (more information in Supplementary Table 1). Additionally, participants with missing exposure or covariate data (*n* = 1511) were excluded, leaving 52,247 participants for analysis in the present prospective cohort study (Supplementary Fig. 1).

Establishment of the cohort was approved by relevant scientific ethics committees and the Danish Data Protection Agency, and all participants gave written informed consent.

### Source-specific nitrate and nitrite intakes

Participants, prior to their first study visit, completed a validated semi-quantitative food frequency questionnaire (FFQ) where they reported their usual intake of 192 food and beverages over the preceding 12 months [26–28]. A detailed methodology for the calculation of nitrate and nitrite intakes has been documented earlier [29]. In brief, nitrate and nitrite intakes, except for tap water, were derived using two comprehensive databases [9, 17] and from government analyses as part of national monitoring programmes. Preference was given to values from Danish sources. To compute nitrate/nitrite intakes, we multiplied the reported consumption quantity of each food item (g/day) by its designated median nitrate/nitrite value (mg/g), adjusting for a 50% decrease in nitrate content for boiled vegetables.

For tap water nitrate assessment, we utilized the public national geodatabase Jupiter [30]. By spatially linking this data with the addresses of the cohort participants, individual-level nitrate consumption from tap water for each participant between 1978 and 2016 was estimated. Comprehensive details have been outlined in a previous publication [29]. To estimate baseline intakes of tap water-sourced nitrate, intakes of tap water were summed from the FFQ (considering the consumption of tap water, tea, coffee, and water added to fruit syrup) and multiplied by the time-weighted average of the nitrate concentration at every address each cohort participant lived at in the 12 months prior to their enrolment into the study. As constituents in tea, coffee, and fruit syrup might hinder the formation of NOCs [14], we additionally examine associations for intakes of tap water itself.

Food and beverages were sorted into four primary categories: tap water, foods from plant sources (fruits, vegetables, legumes, and wholegrains), foods from animal sources (red meat, poultry, processed meat, offal, dairy, eggs, fish, and other seafood), and other sources (alcoholic beverages and discretionary foods) [29]. Given that vegetables are the predominant dietary nitrate source [9], for relevant public health recommendations, we also looked specifically at vegetable nitrate/nitrite. Furthermore, we distinguished between inherent versus added nitrate/nitrite in animal-sourced foods, examining ‘naturally occurring animal-sourced nitrate/nitrite’ and ‘additive permitted meat-sourced nitrate/nitrite’ (i.e., bacon, ham, salami, sausage, liver paste, and other processed meats), separately.

### Mortality outcomes

Information regarding the vital status and date of death of each participant was acquired from the Civil Registration System while data concerning the cause of death was obtained from the National Death Register. Cancer-related mortality was defined as a death with cancer (ICD-10: C00-C97) listed as the primary cause while CVD-related mortality was defined as a death with CVD (ICD-10: I00-I99) listed as the primary cause.

### Covariates

Upon enrollment into the Danish Diet, Cancer, and Health Study, participants provided information regarding various demographic and lifestyle factors. Specifically, participants reported their age, sex, smoking status (i.e., current, former, or never), and smoking history (i.e., packyears). Additionally, participants reported their leisure-time physical activity in winter and summer (number of hours per week which was converted to total daily metabolic equivalent score; MET) in a self-administered questionnaire. Height and weight were objectively measured at baseline and body mass index (BMI) was calculated in kg/m². Data pertaining to the participants’ education level (≤ 7 years, 8–12 years, or ≥ 12 years) and living situation (living with a partner/single) were obtained from the Education Registry and the Civil Registration System, respectively. Intakes of alcohol (g/d), total polyphenols (mg/d; calculated using the Phenol-Explorer database [31]), folate (µg/d), vitamin C (µg/d), and vitamin E (mg α tocopherol equivalents/d) were estimated from the FFQ. Prevalent chronic kidney disease (CKD) and chronic obstructive pulmonary disease (COPD) were defined as a record of the respective disease in either the DNPR or the RUKS registry, while prevalent diabetes was defined as a record of either type 1 or type 2 diabetes in the RUKS registry (see Supplementary Table 1 for more information).

### Statistical analysis

Baseline characteristics of the 52,247 cohort participants are presented for the whole cohort as well as for the lowest and highest quintiles of plant-sourced nitrate, animal-sourced nitrate, and water-sourced nitrate intakes. Participants’ time-to-event was calculated from the date of enrolment until the date of death, emigration, or end of follow-up (31 December 2020), whichever came first. To allow for non-linear relationships between exposures and outcomes, continuous exposure variables were fitted as restricted cubic splines (with 4 knots placed at the 5th, 35th, 65th, and 95th percentiles and the median intake in the lowest quintile taken to be the reference) within separate Cox proportional hazards models for each exposure/outcome combination. The resulting HRs (95% CIs) were graphed with x-axes truncated at 3 standard deviations above the mean for visual simplicity. In tables, HRs and 95% CIs are presented for the median intake in each quintile. No violations in proportional hazards assumptions, assessed via visual inspection of the parallelism of log-log plots of the survival function versus time, were observed. To examine consistency of associations, analyses were stratified by sex (male vs. female), smoking status (ever vs. never), and intakes of vitamin C, vitamin E, folate, and polyphenols (tertile 3 vs. tertile 1 for all). Covariates were chosen *a priori* using prior knowledge of potential confounders of nitrate intake and premature death. Three models of adjustment were used: Model 1 included age and sex; Model 2 included age, sex, BMI, smoking status, smoking packyears, alcohol consumption, education level, physical activity level and living situation; Model 3 adjusted for the covariates in Model 2 plus intakes of (a) red meat, processed meat, poultry, dairy, fish, sugar and confectionary, soft drinks, refined grains, coffee, and tea when plant-sourced nitrate or nitrite were the exposures of interest, (b) wholegrains, refined grains, vegetables, fruits, vegetable oils, sugar and confectionary, soft drinks, coffee, and tea when animal-sourced nitrate or nitrite were the exposures of interest and (c) wholegrains, refined grains, red meat, processed meat, poultry, dairy, fish, vegetables, fruits, vegetable oils, sugar and confectionary, and soft drinks when water-sourced nitrate was the exposure of interest. We used the ‘all-components model’ approach for Model 3, i.e. adjustment for food groups excluding the exposure food group, as it provides unbiased estimates compared to other methods of energy adjustment [32] and accounts for underlying dietary patterns. In a sensitivity analysis, we additionally adjusted for the exposure food group. All continuous covariates were modelled with restricted cubic splines. As nitrate samples in private wells are sparse, in a sensitivity analysis we excluded participants who were suppled by a private well in the 12 months prior to baseline. To quantify effect estimates on the absolute scale, we furthermore analysed associations between source-dependent nitrate intakes and all-cause mortality using regression analyses of restricted mean survival time based on pseudo-observations [33]. Differences in restricted mean survival time (RMST) at 20 years, according to the different exposure variables, were estimated (Model 3 adjustment). Similar to the Cox regression models, exposure variables were specified as restricted cubic splines and contrast estimates with 95% confidence intervals for the median intake in each quintile compared to the median intake in the first quintile are presented. In a sensitivity analysis we analysed nitrate from drinking water as a time-varying exposure variable, expressing average 15-year exposure. At each point in time, the exposure was assessed by taking the cumulative average concentration of nitrate in drinking water supplied to the addresses where each participant had lived over the previous fifteen years and modelled with a restricted cubic spline. For this analysis, a complete case approach was applied and, as exposure information was only available until 2016, end of follow-up was 31 December 2016. Analyses were undertaken using R statistics (R Core Team, 2022) and SAS 9.4 (SAS Institute, Cary NC). Statistical significance was set at *p* ≤ 0.05 (two-tailed) for all tests.

### Role of funding source

The funding source had no role in study design, preparation of this manuscript, or decision to submit the paper for publication.

## Results

The 52,247 study participants had a median [IQR] age of 56 [52–60] years at study entry, and a median [IQR] follow-up time of 24 [22 – 25] years. Over a maximum follow-up of ∼ 27 years, 16,883 individuals died from any cause, 3,358 died with the primary cause of death attributed to CVD and 7,093 died with the primary cause of death attributed to cancer.

### Baseline characteristics

Compared to participants in the lowest quintile of plant-sourced nitrate intake, those in the highest quintile were more likely to be female, be more physically active, have never smoked, have a higher degree of education, be living with a partner, and were less likely to have COPD but more likely to have diabetes (Table 1). For animal-sourced nitrate, similar, although less stark, differences between the highest and lowest consumers were observed except that participants with the highest intakes were more likely to be male. For water-sourced nitrate, participants with the highest intakes were more likely to be female, more physically active, current smokers, not living with a partner, and have a lower degree of education.

**Table 1:** Baseline characteristics of the study population

Characteristics	Categories source-specific nitrate intakes						
	Total participants	Plant-sourced nitrate (Q1)	Plant-sourced nitrate (Q5)	Animal-sourced nitrate (Q1)	Animal-sourced nitrate (Q5)	Water-sourced nitrate (Q1)	Water-sourced nitrate (Q5)
	(*N* = 52,247)	(*n* = 10,450)	(*n* = 10,450)	(*n* = 10,450)	(*n* = 10,450)	(*n* = 10,450)	(*n* = 10,450)
Plant-sourced nitrate intake, mg/d	44 [31, 60]	22 [18, 26]	77 [70, 88]	36 [24, 51]	53 [39, 70]	42 [30, 57]	45 [31, 61]
Animal-sourced nitrate intake, mg/d	5.9 [4.1, 8.4]	4.6 [3.4, 6.7]	7.1 [4.7, 9.9]	3.0 [2.5, 3.4]	11.1 [9.9, 13.9]	5.8 [4.1, 8.3]	5.9 [4.0, 8.5]
Water-sourced nitrate intake (from all tap water sources), mg/d	3.0 [1.8, 5.0]	2.9 [1.7, 4.9]	3.3 [2.0, 5.2]	3.2 [1.9, 5.2]	3.1 [1.8, 5.0]	1.0 [0.7, 1.3]	8.4 [6.6, 12.4]
**Sociodemographics**							
Age, years	56 [52, 60]	56 [52, 60]	55 [52, 59]	56 [52, 60]	56 [52, 60]	55 [52, 59]	55 [52, 59]
Sex (male)	24,293 (46.5)	5145 (49.2)	4242 (40.6)	3244 (31.0)	5251 (50.2)	5812 (55.6)	4050 (38.8)
MET score	56 [37, 85]	49 [31, 76]	65 [43, 94]	53 [34, 80]	62 [40, 91]	52 [34, 78]	60 [38, 90]
BMI (kg/m^2^)	25 [23, 28]	26 [23, 29]	25 [23, 27]	25 [23, 28]	26 [23, 28]	26 [23, 28]	26 [23, 28]
Smoking status							
Never	18,875 (36.1)	3011 (28.8)	4215 (40.3)	3719 (35.6)	3997 (38.2)	4128 (39.5)	3476 (33.3)
Former	14,645 (28.0)	2419 (23.1)	3360 (32.2)	2689 (25.7)	3043 (29.1)	3078 (29.5)	2726 (26.1)
Current	18,727 (35.8)	5020 (48.0)	2875 (27.5)	4042 (38.7)	3410 (32.6)	3244 (31.0)	4248 (40.7)
Smoking (pack-years)	8 [0, 26]	18 [0, 32]	4 [0, 20]	9 [0, 26]	6 [0, 24]	6 [0, 24]	11 [0, 27]
Education							
≤ 7 years	9382 (18.0)	2613 (25.0)	1355 (13.0)	2168 (20.7)	1835 (17.6)	1760 (16.8)	2185 (20.9)
8–12 years	28,469 (54.5)	6281 (60.1)	4866 (46.6)	6000 (57.4)	5230 (50.0)	5634 (53.9)	5846 (55.9)
≥ 12 years	14,396 (27.6)	1556 (14.9)	4229 (40.5)	2282 (21.8)	3385 (32.4)	3056 (29.2)	2419 (23.1)
Marital status (single)	13,570 (26.0)	3345 (32.0)	2867 (27.4)	3166 (30.3)	2939 (28.1)	2110 (20.2)	3131 (30.0)
**Co-morbidities**							
CKD	154 (0.3)	28 (0.3)	25 (0.2)	35 (0.3)	25 (0.2)	25 (0.2)	34 (0.3)
COPD	2013 (3.9)	534 (5.1)	337 (3.2)	482 (4.6)	360 (3.4)	346 (3.3)	486 (4.7)
Diabetes	637 (1.2)	93 (0.9)	183 (1.8)	119 (1.1)	136 (1.3)	93 (0.9)	160 (1.5)
**Dietary intakes**							
Energy (kcal/d)	2269 [1876, 2717]	1945 [1597, 2339]	2571 [2167, 3056]	1815 [1538, 2146]	2660 [2255, 3143]	2278 [1894, 2703]	2264 [1855, 2723]
Vegetable (g/d)	310 [232, 403]	184 [143, 223]	475 [403, 565]	257 [186, 344]	358 [276, 457]	306 [231, 390]	313 [231, 416]
Fruit (g/d)	170 [93, 280]	88 [40, 153]	283 [178, 432]	140 [68, 246]	220 [133, 347]	158 [86, 264]	177 [99, 296]
Wholegrain (g/d)	122 [81, 169]	104 [66, 148]	153 [104, 204]	112 [68, 163]	135 [99, 186]	119 [80, 165]	125 [84, 171]
Red meat (g/d)	81 [58, 111]	75 [55, 100]	81 [56, 116]	56 [42, 70]	101 [73, 138]	87 [63, 117]	77 [56, 107]
Poultry (g/d)	18 [10, 27]	12 [7, 20]	22 [13, 35]	13 [7, 21]	21 [13, 33]	18 [10, 27]	18 [10, 28]
Processed meat (g/d)	22 [12, 35]	22 [13, 37]	19 [10, 33]	16 [8, 26]	25 [14, 39]	23 [14, 37]	21 [11, 35]
Fish (g/d)	38 [25, 55]	29 [19, 42]	47 [32, 68]	28 [19, 41]	47 [32, 67]	37 [25, 53]	38 [25, 55]
Dairy (g/d)	306 [167, 570]	263 [121, 547]	355 [215, 615]	138 [75, 285]	536 [380, 795]	312 [169, 577]	306 [166, 567]
Sugar and confectionery (g/d)	49 [29, 81]	42 [23, 72]	53 [32, 87]	41 [22, 70]	56 [34, 92]	47 [28, 75]	51 [29, 88]
Tea (g/d)	86 [3, 500]	16 [3, 200]	200 [16, 500]	86 [3, 500]	157 [16, 500]	29 [3, 200]	157 [7, 500]
Coffee (g/d)	900 [500, 1300]	900 [500, 1300]	500 [500, 900]	900 [500, 1300]	900 [500, 1300]	500 [500, 900]	900 [500, 1300]
Soft drink (g/d)	16 [3, 45]	16 [3, 86]	7 [0, 29]	7 [0, 29]	16 [3, 57]	16 [3, 86]	16 [3, 33]
Vegetable oils (g/d)	5 [1, 9]	1 [1, 3]	9 [5, 13]	2 [1, 6]	5 [2, 11]	4 [1, 9]	5 [1, 9]
Alcohol (g/d)	13 [6, 31]	12 [4, 32]	13 [6, 29]	11 [3, 26]	13 [6, 30]	14 [7, 32]	12 [5, 24]
Polyphenols (mg/d)	1609 [1266, 1951]	1430 [1091, 1771]	1769 [1430, 2158]	1523 [1167, 1866]	1684 [1347, 2056]	1357 [1036, 1708]	1808 [1458, 2171]
Vitamin C (mg/d)	125 [87, 184]	75 [53, 116]	185 [141, 259]	109 [71, 166]	152 [109, 216]	116 [82, 169]	131 [90, 194]
Vitamin E (mg α-TE/d)	12 [8, 18]	9 [6, 14]	15 [11, 21]	10 [7, 16]	14 [10, 20]	11 [8, 17]	13 [9, 18]
Folic acid (µg/d)	368 [290, 463]	273 [219, 350]	473 [398, 567]	301 [235, 388]	446 [363, 542]	350 [276, 440]	381 [298, 481]

Data expressed as median [IQR] or n (%), unless otherwise stated

BMI, body mass index; CKD, chronic kidney disease; COPD, common obstructive pulmonary disease; CVD, cardiovascular disease; MET, metabolic equivalent (determined from physical activity questionnaire)

### Associations between plant- and vegetable-sourced nitrate and nitrite intake and mortality

**Fig. 1:**
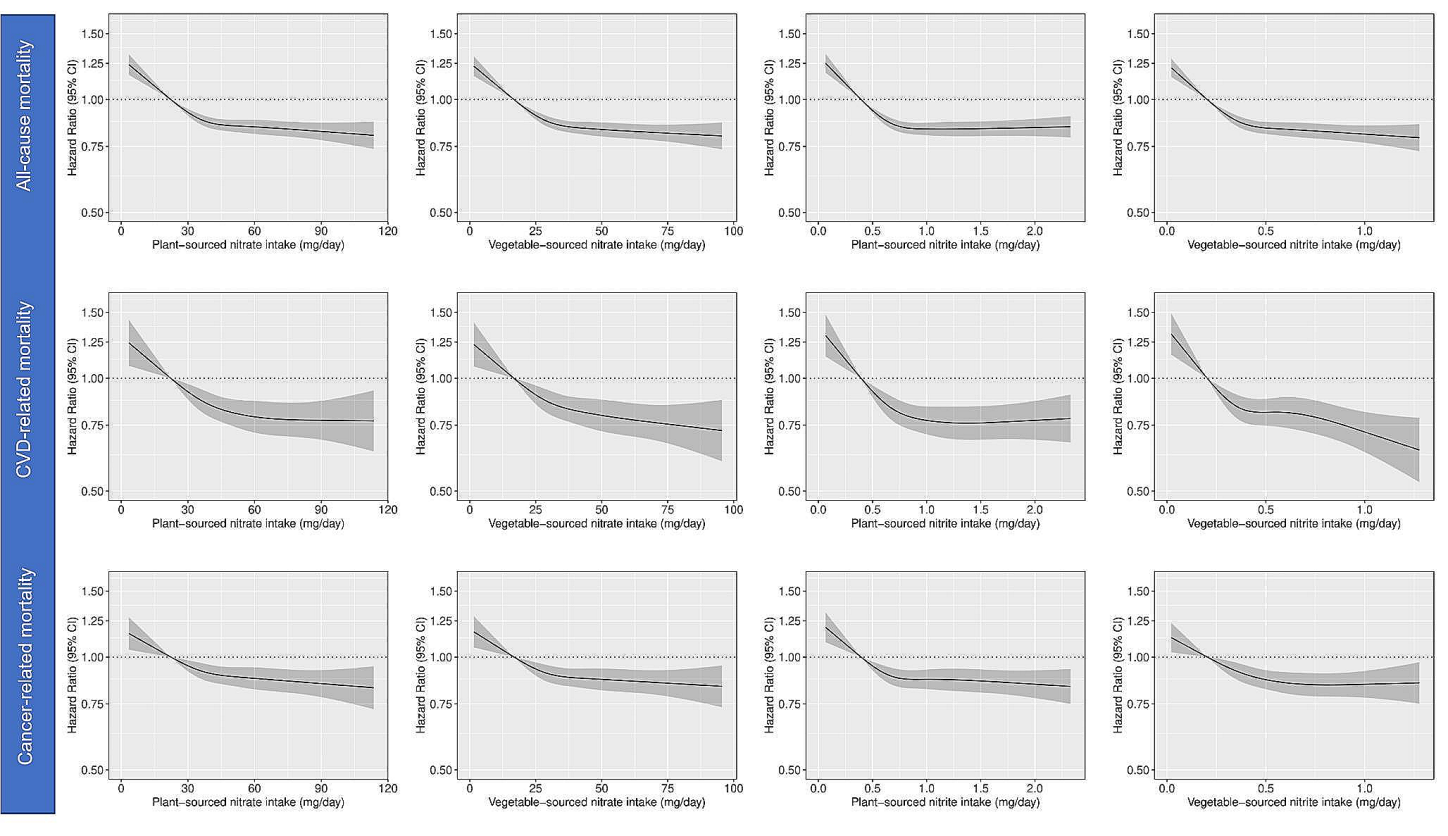
Cubic spline curves depicting the association between plant- and vegetable-sourced nitrate and nitrite intakes and all-cause mortality, cardiovascular (CVD)-related mortality and cancer-related mortality in participants of the Danish Diet Cancer and Health cohort (*n* = 52,247). Hazard ratios and 95% CI’s are based on Cox proportional hazards models adjusted for age, sex, BMI, smoking status, smoking packyears, alcohol consumption, education level, physical activity level, living situation, and intakes of red meat, processed meat, poultry, dairy, fish, sugar and confectionary, soft drinks, refined grains, coffee, and tea (Model 3) and are comparing the specific level of the exposure (horizontal axis) to the median intake for participants in the lowest intake quintile

Restricted cubic splines show a non-linear inverse association between intakes of plant-sourced nitrate and all-cause mortality, CVD-related mortality, and cancer-related mortality, where inverse associations plateaued around moderate intakes (Fig. 1). Compared to participants in quintile 1 (median intake: 22 mg/d), those in quintile 5 (median intake: 77 mg/d) had a 17% lower risk of all-cause mortality [Model 3 HR_Q5vsQ1_: 0.83 (0.80, 0.87), Table 2], a 22% lower risk of CVD-related mortality [Model 3 HR_Q5vsQ1_: 0.78 (0.70, 0.86), Table 2], and a 14% lower risk of cancer-related mortality [Model 3 HR_Q5vsQ1_: 0.86 (0.80, 0.93), Table 2]. Across all outcomes, significantly lower risks were already observed for participants in quintile 2, compared to those in quintile 1 (Table 2). Comparable non-linear inverse associations were seen when focussing on only vegetable-sourced nitrate (Fig. 1**and** Table 2).

**Table 2:** Hazards ratio of mortality subtypes by quintiles of plant-sourced and vegetable-sourced nitrate and nitrite intake

	Q1	Q2	Q3	Q4	Q5
	*n* = 10,450	*n* = 10,449	*n* = 10,449	*n* = 10,449	*n* = 10,450
**Plant-sourced nitrate**					
Intake (mg/d)	22 [18, 26]	34 [31, 36]	44 [41, 47]	56 [53, 60]	77 [70, 88]
All-cause mortality					
No. events	4292	3661	3175	2961	2794
Model 1	Ref.	0.79 (0.77, 0.81)	0.70 (0.68, 0.72)	0.66 (0.63, 0.68)	0.63 (0.61, 0.66)
Model 2	Ref.	0.91 (0.88, 0.93)	0.87 (0.84, 0.90)	0.86 (0.83, 0.90)	0.86 (0.82, 0.89)
Model 3	Ref.	0.90 (0.87, 0.92)	0.86 (0.83, 0.89)	0.85 (0.81, 0.88)	0.83 (0.80, 0.87)
Cardiovascular disease-related mortality					
No. events	862	762	644	557	533
Model 1	Ref.	0.79 (0.75, 0.84)	0.68 (0.64, 0.73)	0.62 (0.57, 0.67)	0.60 (0.55, 0.66)
Model 2	Ref.	0.91 (0.86, 0.97)	0.86 (0.80, 0.93)	0.84 (0.77, 0.91)	0.84 (0.76, 0.92)
Model 3	Ref.	0.89 (0.83, 0.95)	0.83 (0.77, 0.90)	0.80 (0.73, 0.87)	0.78 (0.70, 0.86)
Cancer-related mortality					
No. events	1740	1502	1371	1297	1183
Model 1	Ref.	0.84 (0.80, 0.87)	0.75 (0.72, 0.79)	0.70 (0.66, 0.74)	0.67 (0.63, 0.71)
Model 2	Ref.	0.93 (0.90, 0.98)	0.90 (0.86, 0.95)	0.89 (0.84, 0.94)	0.87 (0.81, 0.93)
Model 3	Ref.	0.93 (0.89, 0.97)	0.90 (0.85, 0.94)	0.88 (0.83, 0.94)	0.86 (0.80, 0.93)
**Vegetable-sourced nitrate**					
Intake (mg/d)	17 [13, 19]	26 [24, 29]	35 [33, 38]	46 [43, 49]	65 [58, 74]
All-cause mortality					
No. events	4226	3682	3171	2969	2835
Model 1	Ref.	0.81 (0.79, 0.83)	0.72 (0.69, 0.74)	0.67 (0.64, 0.69)	0.65 (0.62, 0.67)
Model 2	Ref.	0.91 (0.89, 0.94)	0.87 (0.84, 0.90)	0.86 (0.82, 0.89)	0.85 (0.81, 0.88)
Model 3	Ref.	0.90 (0.87, 0.92)	0.85 (0.82, 0.88)	0.84 (0.80, 0.87)	0.82 (0.78, 0.86)
Cardiovascular disease-related mortality					
No. events	837	763	646	583	529
Model 1	Ref.	0.81 (0.76, 0.86)	0.70 (0.66, 0.76)	0.64 (0.59, 0.70)	0.61 (0.56, 0.67)
Model 2	Ref.	0.92 (0.87, 0.98)	0.87 (0.81, 0.94)	0.85 (0.78, 0.92)	0.83 (0.76, 0.92)
Model 3	Ref.	0.89 (0.84, 0.95)	0.84 (0.77, 0.90)	0.81 (0.74, 0.88)	0.77 (0.69, 0.85)
Cancer-related mortality					
No. events	1716	1513	1346	1320	1198
Model 1	Ref.	0.85 (0.82, 0.89)	0.77 (0.73, 0.81)	0.72 (0.68, 0.76)	0.69 (0.65, 0.74)
Model 2	Ref.	0.93 (0.89, 0.97)	0.90 (0.86, 0.95)	0.88 (0.83, 0.94)	0.87 (0.81, 0.93)
Model 3	Ref.	0.92 (0.88, 0.96)	0.89 (0.84, 0.94)	0.88 (0.82, 0.93)	0.86 (0.80, 0.92)
**Plant-sourced nitrite**					
Intake (mg/d)	0.4 [0.3, 0.5]	0.6 [0.6, 0.7]	0.8 [0.8, 0.9]	1.0 [1.0, 1.1]	1.5 [1.3, 1.7]
All-cause mortality					
No. events	4465	3524	3078	2883	2933
Model 1	Ref.	0.76 (0.74, 0.78)	0.67 (0.65, 0.69)	0.63 (0.61, 0.65)	0.63 (0.61, 0.66)
Model 2	Ref.	0.88 (0.86, 0.91)	0.85 (0.82, 0.87)	0.84 (0.81, 0.88)	0.85 (0.82, 0.89)
Model 3	Ref.	0.88 (0.86, 0.91)	0.84 (0.81, 0.87)	0.83 (0.80, 0.87)	0.84 (0.80, 0.88)
Cardiovascular disease-related mortality					
No. events	929	687	639	556	547
Model 1	Ref.	0.75 (0.71, 0.80)	0.64 (0.60, 0.69)	0.59 (0.55, 0.64)	0.59 (0.54, 0.64)
Model 2	Ref.	0.88 (0.82, 0.93)	0.82 (0.77, 0.89)	0.81 (0.75, 0.88)	0.81 (0.74, 0.89)
Model 3	Ref.	0.86 (0.81, 0.92)	0.80 (0.74, 0.86)	0.77 (0.70, 0.84)	0.76 (0.69, 0.84)
Cancer-related mortality					
No. events	1793	1510	1314	1250	1226
Model 1	Ref.	0.79 (0.76, 0.83)	0.71 (0.68, 0.75)	0.68 (0.64, 0.72)	0.67 (0.63, 0.71)
Model 2	Ref.	0.91 (0.87, 0.95)	0.88 (0.83, 0.92)	0.88 (0.83, 0.93)	0.87 (0.81, 0.93)
Model 3	Ref.	0.91 (0.87, 0.95)	0.87 (0.83, 0.92)	0.87 (0.82, 0.93)	0.86 (0.81, 0.93)
**Vegetable-sourced nitrite**					
Intake (mg/d)	0.2 [0.2, 0.2]	0.3 [0.3, 0.4]	0.5 [0.4, 0.5]	0.6 [0.6, 0.6]	0.9 [0.8, 1.0]
All-cause mortality					
No. events	4385	3625	3075	2993	2805
Model 1	Ref.	0.81 (0.78, 0.83)	0.71 (0.69, 0.73)	0.68 (0.65, 0.70)	0.66 (0.64, 0.69)
Model 2	Ref.	0.91 (0.88, 0.94)	0.87 (0.84, 0.90)	0.86 (0.83, 0.89)	0.85 (0.82, 0.89)
Model 3	Ref.	0.89 (0.87, 0.92)	0.85 (0.82, 0.88)	0.83 (0.80, 0.87)	0.82 (0.78, 0.86)
Cardiovascular disease-related mortality					
No. events	911	714	606	605	522
Model 1	Ref.	0.78 (0.74, 0.83)	0.69 (0.64, 0.74)	0.66 (0.61, 0.72)	0.63 (0.58, 0.69)
Model 2	Ref.	0.89 (0.84, 0.95)	0.86 (0.80, 0.92)	0.87 (0.80, 0.94)	0.84 (0.77, 0.93)
Model 3	Ref.	0.86 (0.80, 0.92)	0.81 (0.75, 0.88)	0.81 (0.74, 0.89)	0.76 (0.69, 0.85)
Cancer-related mortality					
No. events	1731	1553	1339	1256	1214
Model 1	Ref.	0.86 (0.82, 0.90)	0.77 (0.73, 0.81)	0.71 (0.67, 0.76)	0.69 (0.65, 0.74)
Model 2	Ref.	0.94 (0.90, 0.99)	0.90 (0.85, 0.95)	0.87 (0.82, 0.92)	0.86 (0.81, 0.92)
Model 3	Ref.	0.93 (0.89, 0.97)	0.88 (0.83, 0.93)	0.85 (0.80, 0.91)	0.84 (0.78, 0.91)

Hazard ratios (95% CI) for all-cause, cardiovascular disease-related mortality, and cancer-related mortality during 27 years of follow up, obtained from restricted cubic splines based on Cox proportional hazards models. Model 1 included age and sex; Model 2 included age, sex, BMI, smoking status, smoking packyears, alcohol consumption, education level, physical activity level and living situation; Model 3 adjusted for the covariates in Model 2 plus intakes of red meat, processed meat, poultry, dairy, fish, sugar and confectionary, soft drinks, refined grains, coffee, and tea

Exposure intakes are presented as median [IQR]

Associations for intakes of plant-sourced and vegetable-sourced nitrite were almost identical to those observed for nitrate [Model 3 HR_Q5vsQ1_ for plant-sourced nitrite; all-cause mortality 0.84 (0.80, 0.88); CVD-related mortality 0.76 (0.69, 0.84); cancer-related mortality 0.86 (0.81, 0.93), Table 2]. In a sensitivity analysis, associations with all-cause mortality remained when we additionally adjusted for intakes of all plant-based foods [HR_Q5vsQ1_ for plant-sourced nitrate: 0.87 (0.80, 0.93); plant-sourced nitrite 0.87 (0.82, 0.94); vegetable-sourced nitrate: 0.86 (0.80, 0.91); and vegetable-sourced nitrite 0.86 (0.81, 0.91)].

Over a 20-year period, individuals with the highest plant- and vegetable-sourced nitrate intakes are expected to live, on average, 6 months longer [Model 3 RMST_Q5vsQ1_ (95% CI); plant: 6 (5, 7); vegetable: 6 (5, 8)] than individuals with the lowest intakes.

### Associations between animal-sourced nitrate and nitrite intake and mortality

**Fig. 2:**
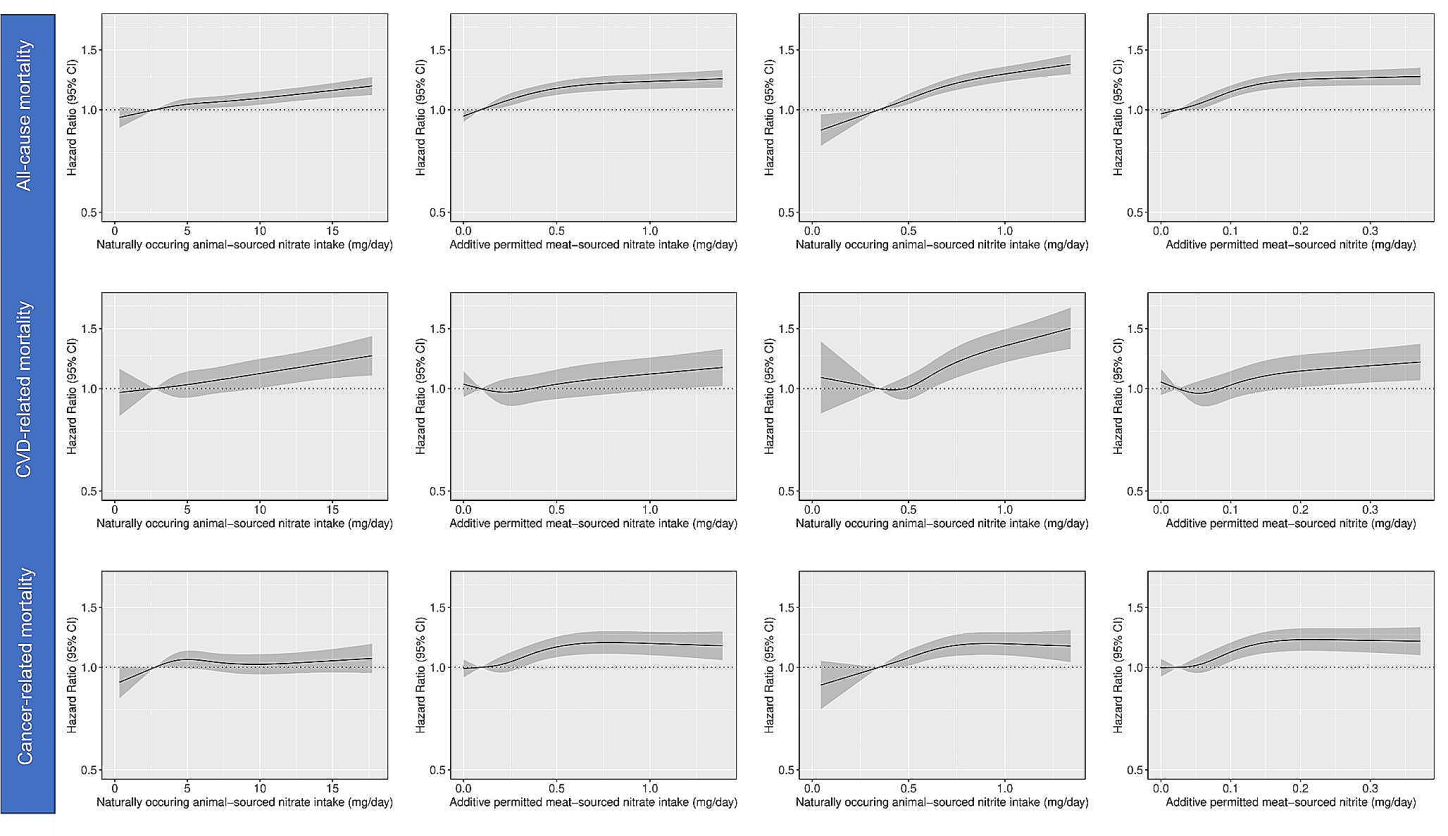
Cubic spline curves depicting the association between naturally occurring animal- and processed meat-sourced nitrate and nitrite intakes and all-cause mortality, cardiovascular (CVD)-related mortality and cancer-related mortality in participants of the Danish Diet Cancer and Health cohort (*n* = 52,247). Hazard ratios and 95% CI’s are based on Cox proportional hazards models adjusted for age, sex, BMI, smoking status, smoking packyears, alcohol consumption, education level, physical activity level, living situation and intakes of wholegrains, refined grains, vegetables, fruits, vegetable oils, sugar and confectionary, soft drinks, refined grains, coffee, and tea (Model 3) and are comparing the specific level of the exposure (horizontal axis) to the median intake for participants in the lowest intake quintile

Intakes of naturally occurring animal-sourced nitrate were directly associated with all-cause and CVD-related, but not cancer-related, mortality (Fig. 2). Participants with the highest (median intake: 10.7 mg/d), compared to those with the lowest (median intake: 2.7 mg/d) intakes had a 9% higher risk of all-cause mortality [Model 3 HR_Q5vsQ1_: 1.09 (1.04, 1.14), Table 3] and a 12% higher risk of CVD-related mortality [Model 3 HR_Q5vsQ1_: 1.12 (1.01, 1.24), Table 3], but no higher risk of cancer-related mortality [Model 3 HR_Q5vsQ1_: 1.02 (0.95, 1.10), Table 3]. Intakes of naturally occurring animal-sourced nitrite were directly associated with all outcomes (Fig. 2). Participants with the highest (median intake: 0.9 mg/d), compared to those with the lowest (median intake: 0.3 mg/d) intakes had a 25% higher risk of all-cause mortality [Model 3 HR_Q5vsQ1_: 1.25 (1.19, 1.31), Table 3], a 29% higher risk of CVD-related mortality [Model 3 HR_Q5vsQ1_: 1.29 (1.15, 1.44), Table 3], and an 18% higher risk of cancer-related mortality [Model 3 HR_Q5vsQ1_: 1.18 (1.09, 1.27), Table 3]. In a sensitivity analysis, associations with all-cause mortality remained for naturally occurring animal-sourced nitrite, but not nitrate, when we additionally adjusted for intakes of all animal-based foods [HR_Q5vsQ1_ naturally occurring animal-sourced nitrate: 1.02 (0.97, 1.08); naturally occurring animal-sourced nitrite 1.18 (1.11, 1.25)].

**Table 3:** Hazards ratio of mortality subtypes by quintiles of naturally occurring and additive permitted meat-sourced nitrate and nitrite intake

	Q1	Q2	Q3	Q4	Q5
	*n* = 10,450	*n* = 10,449	*n* = 10,449	*n* = 10,449	*n* = 10,450
**Naturally occurring animal-sourced nitrate**					
Intake (mg/d)	2.7 [2.3, 3.1]	4.0 [3.7, 4.3]	5.4 [5.0, 5.9]	7.5 [7.0, 8.1]	10.7 [9.5, 13.4]
All-cause mortality					
No. events	3431	3441	3389	3221	3401
Model 1	Ref.	0.95 (0.93, 0.98)	0.91 (0.87, 0.94)	0.87 (0.84, 0.90)	0.87 (0.84, 0.91)
Model 2	Ref.	0.98 (0.95, 1.01)	0.97 (0.93, 1.01)	0.97 (0.93, 1.01)	0.99 (0.95, 1.03)
Model 3	Ref.	1.02 (0.99, 1.06)	1.04 (1.00, 1.08)	1.06 (1.02, 1.10)	1.09 (1.04, 1.14)
Cardiovascular disease-related mortality					
No. events	646	676	710	619	707
Model 1	Ref.	0.95 (0.89, 1.02)	0.91 (0.84, 1.00)	0.88 (0.81, 0.96)	0.90 (0.82, 0.99)
Model 2	Ref.	0.97 (0.91, 1.04)	0.96 (0.88, 1.05)	0.97 (0.89, 1.06)	1.01 (0.92, 1.10)
Model 3	Ref.	1.01 (0.95, 1.09)	1.03 (0.94, 1.13)	1.07 (0.97, 1.17)	1.12 (1.01, 1.24)
Cancer-related mortality					
No. events	1453	1487	1443	1353	1357
Model 1	Ref.	0.99 (0.95, 1.04)	0.95 (0.90, 1.01)	0.88 (0.83, 0.93)	0.85 (0.80, 0.91)
Model 2	Ref.	1.02 (0.97, 1.06)	1.01 (0.95, 1.07)	0.97 (0.91, 1.03)	0.95 (0.89, 1.02)
Model 3	Ref.	1.04 (1.00, 1.09)	1.05 (0.99, 1.12)	1.03 (0.97, 1.10)	1.02 (0.95, 1.10)
**Additive permitted meat-sourced nitrate**					
Intake (mg/d)	0.09 [0.06, 0.12]	0.18 [0.16, 0.20]	0.28 [0.26, 0.31]	0.43 [0.38, 0.48]	0.73 [0.62, 0.95]
All-cause mortality					
No. events	2929	2949	3270	3602	4133
Model 1	Ref.	1.02 (0.99, 1.05)	1.08 (1.03, 1.12)	1.18 (1.14, 1.23)	1.33 (1.27, 1.39)
Model 2	Ref.	1.03 (1.00, 1.06)	1.06 (1.01, 1.10)	1.09 (1.05, 1.13)	1.13 (1.08, 1.18)
Model 3	Ref.	1.04 (1.01, 1.07)	1.09 (1.04, 1.13)	1.14 (1.09, 1.19)	1.19 (1.14, 1.25)
Cardiovascular disease-related mortality					
No. events	567	537	645	737	872
Model 1	Ref.	0.98 (0.92, 1.05)	1.01 (0.92, 1.11)	1.10 (1.01, 1.21)	1.26 (1.14, 1.39)
Model 2	Ref.	0.98 (0.91, 1.05)	0.98 (0.89, 1.07)	1.00 (0.91, 1.09)	1.04 (0.94, 1.16)
Model 3	Ref.	0.98 (0.91, 1.05)	0.98 (0.89, 1.08)	1.01 (0.92, 1.12)	1.07 (0.96, 1.19)
Cancer-related mortality					
No. events	1228	1247	1376	1520	1722
Model 1	Ref.	1.02 (0.97, 1.07)	1.08 (1.02, 1.16)	1.21 (1.14, 1.29)	1.36 (1.27, 1.46)
Model 2	Ref.	1.01 (0.96, 1.06)	1.04 (0.98, 1.11)	1.10 (1.04, 1.17)	1.15 (1.07, 1.24)
Model 3	Ref.	1.01 (0.97, 1.06)	1.05 (0.99, 1.12)	1.12 (1.05, 1.20)	1.18 (1.10, 1.28)
**Naturally occurring animal-sourced nitrite**					
Intake (mg/d)	0.3 [0.3, 0.4]	0.5 [0.4, 0.5]	0.6 [0.5, 0.6]	0.7 [0.6, 0.7]	0.9 [0.8, 1.1]
All-cause mortality					
No. events	2894	3073	3290	3505	4121
Model 1	Ref.	1.00 (0.97, 1.03)	1.03 (0.99, 1.07)	1.10 (1.06, 1.14)	1.21 (1.16, 1.26)
Model 2	Ref.	1.02 (0.99, 1.05)	1.05 (1.01, 1.08)	1.08 (1.04, 1.12)	1.13 (1.08, 1.18)
Model 3	Ref.	1.06 (1.02, 1.09)	1.11 (1.07, 1.15)	1.17 (1.12, 1.22)	1.25 (1.19, 1.31)
Cardiovascular disease-related mortality					
No. events	528	558	654	698	920
Model 1	Ref.	0.96 (0.90, 1.02)	1.00 (0.92, 1.08)	1.11 (1.01, 1.21)	1.29 (1.17, 1.43)
Model 2	Ref.	0.97 (0.91, 1.04)	1.00 (0.92, 1.08)	1.06 (0.97, 1.16)	1.16 (1.05, 1.29)
Model 3	Ref.	1.00 (0.93, 1.07)	1.05 (0.96, 1.14)	1.14 (1.04, 1.26)	1.29 (1.15, 1.44)
Cancer-related mortality					
No. events	1226	1347	1386	1510	1624
Model 1	Ref.	1.02 (0.98, 1.07)	1.06 (1.01, 1.12)	1.13 (1.06, 1.20)	1.19 (1.11, 1.27)
Model 2	Ref.	1.03 (0.99, 1.08)	1.06 (1.00, 1.12)	1.09 (1.03, 1.16)	1.10 (1.03, 1.18)
Model 3	Ref.	1.05 (1.00, 1.10)	1.09 (1.03, 1.16)	1.14 (1.07, 1.22)	1.18 (1.09, 1.27)
**Additive permitted meat-sourced nitrite**					
Intake (mg/d)	0.02 [0.02, 0.03]	0.05 [0.04, 0.05]	0.07 [0.07, 0.08]	0.11 [0.10 0.12]	0.19 [0.16, 0.24]
All-cause mortality					
No. events	2861	2982	3342	3530	4168
Model 1	Ref.	1.02 (0.99, 1.05)	1.09 (1.04, 1.13)	1.22 (1.17, 1.27)	1.40 (1.34, 1.46)
Model 2	Ref.	1.02 (0.99, 1.05)	1.05 (1.01, 1.10)	1.11 (1.07, 1.16)	1.17 (1.12, 1.23)
Model 3	Ref.	1.03 (1.00, 1.06)	1.08 (1.03, 1.13)	1.15 (1.10, 1.20)	1.22 (1.17, 1.28)
Cardiovascular disease-related mortality					
No. events	546	542	667	712	891
Model 1	Ref.	0.98 (0.91, 1.05)	1.03 (0.94, 1.13)	1.17 (1.07, 1.28)	1.37 (1.24, 1.52)
Model 2	Ref.	0.97 (0.91, 1.04)	0.98 (0.89, 1.08)	1.03 (0.94, 1.13)	1.11 (1.00, 1.23)
Model 3	Ref.	0.97 (0.90, 1.04)	0.98 (0.89, 1.08)	1.04 (0.95, 1.15)	1.12 (1.00, 1.25)
Cancer-related mortality					
No. events	1205	1275	1389	1494	1730
Model 1	Ref.	1.02 (0.98, 1.07)	1.10 (1.03, 1.17)	1.24 (1.17, 1.32)	1.40 (1.31, 1.50)
Model 2	Ref.	1.01 (0.96, 1.06)	1.05 (0.98, 1.11)	1.12 (1.05, 1.19)	1.18 (1.10, 1.27)
Model 3	Ref.	1.01 (0.96, 1.06)	1.05 (0.99, 1.12)	1.13 (1.06, 1.21)	1.20 (1.12, 1.30)

Hazard ratios (95% CI) for all-cause, cardiovascular disease-related mortality, and cancer-related mortality during 27 years of follow up, obtained from restricted cubic splines based on Cox proportional hazards models. Model 1 included age and sex; Model 2 included age, sex, BMI, smoking status, smoking packyears, alcohol consumption, education level, physical activity level and living situation; Model 3 adjusted for the covariates in Model 2 plus intakes of wholegrains, refined grains, vegetables, fruits, vegetable oils, sugar and confectionary, soft drinks, refined grains, coffee, and tea

Exposure intakes are presented as median [IQR]

Intakes of nitrate from additive permitted meat sources were non-linearly directly associated with all-cause and cancer-related mortality (Fig. 2). Participants with the highest (median intake: 0.7 mg/d), compared to those with the lowest (median intake: 0.1 mg/d), intakes had a 19% higher risk of all-cause mortality [Model 3 HR_Q5vsQ1_: 1.19 (1.14, 1.25), Table 3], and an 18% higher risk of cancer-related mortality [Model 3 HR_Q5vsQ1_: 1.18 (1.10, 1.28), Table 3]. Similar associations were seen when looking at intakes of nitrite from additive permitted meat sources (Fig. 2 and Table 3), although the two exposures (additive permitted meat-sourced nitrate and nitrite) were very highly correlated (ρ = 0.95). In a sensitivity analysis, associations with all-cause mortality remained for additive permitted meat-sourced nitrite, but not nitrate, when we additionally adjusted for intakes of processed meat [HR_Q5vsQ1_ additive permitted meat-sourced nitrate: 1.05 (0.92, 1.20); additive permitted meat -sourced nitrite 1.22 (1.06, 1.39)].

Over a 20-year period, individuals with the highest naturally occurring animal-sourced nitrate and nitrite intakes are expected to live on average as long those with the lowest intake [Model 3 RMST_Q5vsQ1_: 0 (-2, 1)] and 4 months less [Model 3 RMST_Q5vsQ1_: -4 (-5, -3)], respectively, than those with the lowest intakes. For additive permitted meat-sourced nitrate and nitrite, individuals with the highest intakes are expected to live on average 4 months less [Model 3 RMST_Q5vsQ1_ (95% CI); nitrate: -4 (-5, -3); nitrite: -4 (-6, -3)] than individuals with the lowest intakes.

### Associations between tap water-sourced nitrate intake and mortality

#### Baseline water nitrate intake

**Fig. 3:**
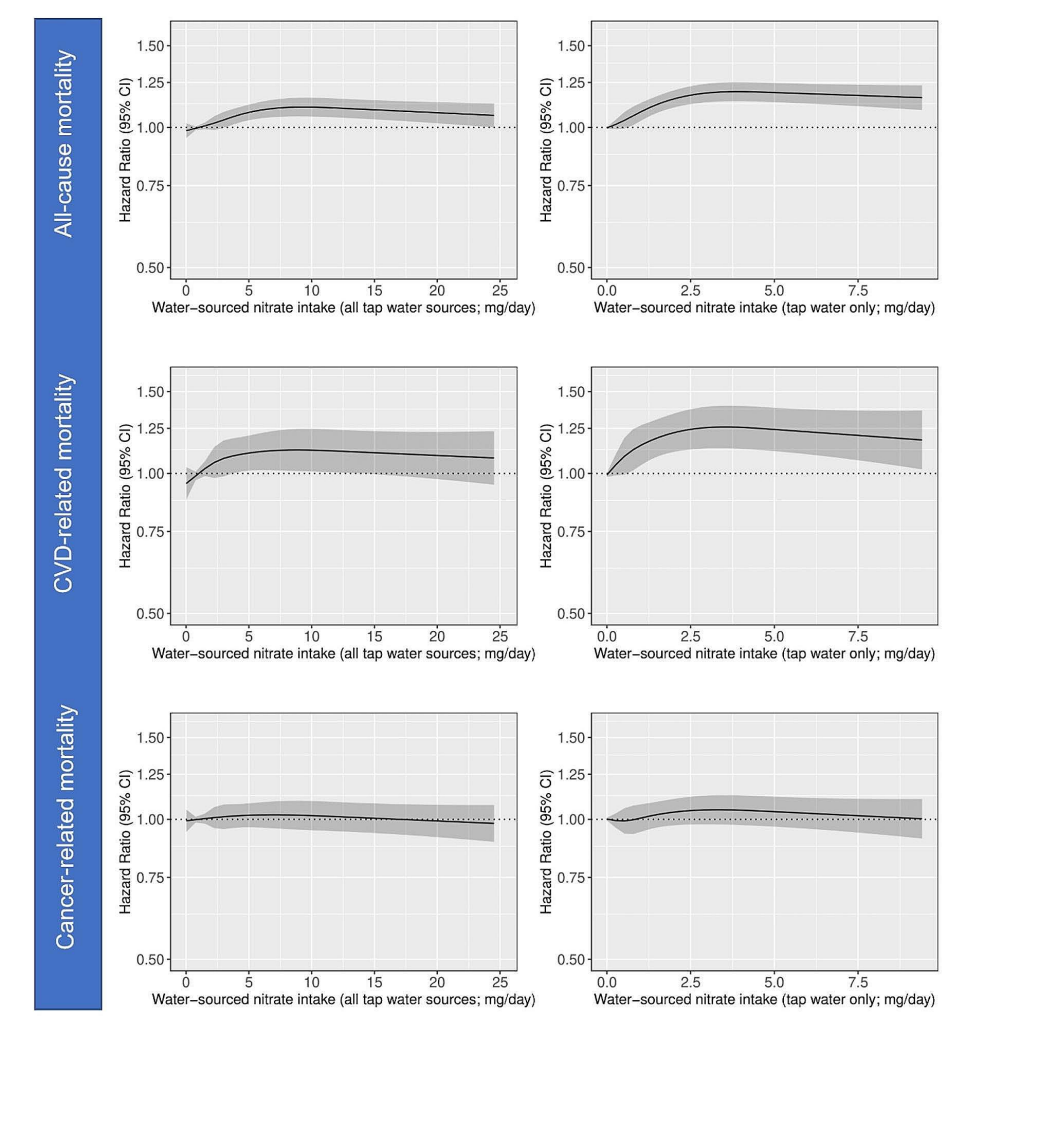
Cubic spline curves depicting the association between water-sourced nitrate intake from (1) all tap water sources (tap water, tea, coffee and tap water added to fruit syrup) and (2) tap water only and all-cause mortality, cardiovascular (CVD)-related mortality and cancer-related mortality in participants of the Danish Diet Cancer and Health cohort (*n* = 52,247). Hazard ratios and 95% CIs are based on Cox proportional hazards models adjusted for age, sex, BMI, smoking status, smoking packyears, alcohol consumption, education level, physical activity level, living situation, and intakes of wholegrains, refined grains, red meat, processed meat, poultry, dairy, fish, vegetables, fruits, vegetable oils, sugar and confectionary, and soft drinks (Model 3) and are comparing the specific level of the exposure (horizontal axis) to the median intake for participants in the lowest intake quintile

A higher consumption of nitrate from all tap water sources at baseline was non-linearly directly associated with a higher risk of all-cause and CVD-related mortality, but not cancer-related mortality (Fig. 3). Participants with the highest intakes (median intake: 8.4 mg/d), compared to those with the lowest (median intake: 1.0 mg/d), had an 11% higher risk of all-cause mortality [Model 3 HR_Q5vsQ1_: 1.11 (1.06, 1.16), Table 4] and a 12% higher risk of CVD-related mortality [Model 3 HR_Q5vsQ1_: 1.12 (1.01, 1.24), Table 4]. When the exposure was restricted to tap water only (that is, not including tap water added to tea, coffee or fruit syrup), associations were stronger (Fig. 3); after adjusting for demographic, lifestyle, and dietary confounders, participants with the highest intakes (median: 3.3 mg/d), compared to those with the lowest intakes (median: 0 mg/d), had a 19% higher risk of all-cause mortality [Model 3 HR_Q5vsQ1_: 1.19 (1.14, 1.25), Table 4] and a 26% higher risk of CVD-related mortality [Model 3 HR_Q5vsQ1_: 1.26 (1.13, 1.40), Table 4]. Associations were unchanged when we additionally adjusted for tap water intake [all-cause mortality: HR_Q5vsQ1_: 1.19 (1.13, 1.25)] or when analyses were restricted to the 52,002 participants who were only supplied by a public well in the 12 months prior to baseline [all-cause mortality: Model 3 HR_Q5vsQ1_: 1.19 (1.14, 1.25)].

**Table 4:** Hazards ratio of mortality subtypes by quintiles of tap water-sourced nitrate intake

	Q1	Q2	Q3	Q4	Q5
	*n* = 10,450	*n* = 10,449	*n* = 10,449	*n* = 10,449	*n* = 10,450
**Water-sourced nitrate from all tap water sources (tap water, tea, coffee, and water added to fruit syrup)**					
Intake (mg/d)	1.0 [0.7, 1.3]	2.0 [1.8, 2.3]	3.0 [2.8, 3.3]	4.4 [4.0, 5.0]	8.4 [6.6, 12.4]
All-cause mortality					
No. events	3166	3291	3399	3500	3527
Model 1	Ref.	1.01 (0.98, 1.04)	1.05 (1.01, 1.09)	1.12 (1.08, 1.16)	1.22 (1.17, 1.28)
Model 2	Ref.	1.00 (0.97, 1.03)	1.01 (0.97, 1.05)	1.03 (0.99, 1.07)	1.07 (1.02, 1.12)
Model 3	Ref.	1.02 (0.99, 1.05)	1.04 (1.00, 1.08)	1.07 (1.03, 1.11)	1.11 (1.06, 1.16)
Cardiovascular disease-related mortality					
No. events	617	692	657	703	689
Model 1	Ref.	1.04 (0.97, 1.11)	1.09 (0.99, 1.19)	1.16 (1.06, 1.26)	1.26 (1.14, 1.39)
Model 2	Ref.	1.02 (0.96, 1.10)	1.04 (0.96, 1.14)	1.06 (0.98, 1.16)	1.09 (0.98, 1.21)
Model 3	Ref.	1.05 (0.98, 1.12)	1.08 (0.99, 1.18)	1.10 (1.01, 1.20)	1.12 (1.01, 1.24)
Cancer-related mortality					
No. events	1362	1415	1428	1457	1431
Model 1	Ref.	1.02 (0.97, 1.06)	1.04 (0.98, 1.11)	1.08 (1.02, 1.14)	1.14 (1.06, 1.22)
Model 2	Ref.	0.99 (0.95, 1.04)	0.99 (0.94, 1.06)	1.00 (0.94, 1.06)	1.00 (0.93, 1.07)
Model 3	Ref.	1.01 (0.96, 1.05)	1.01 (0.95, 1.08)	1.02 (0.96, 1.08)	1.02 (0.95, 1.10)
**Water-sourced nitrate (tap water only)**					
Intake (mg/d)	0.0 [0.0, 0.1]	0.3 [0.2, 0.4]	0.8 [0.6, 0.9]	1.5 [1.1, 1.8]	3.3 [2.6, 4.9]
All-cause mortality					
No. events	3670	3167	3258	3325	3463
Model 1	Ref.	0.89 (0.87, 0.92)	0.86 (0.82, 0.90)	0.94 (0.90, 0.98)	1.06 (1.02, 1.11)
Model 2	Ref.	0.99 (0.96, 1.02)	1.01 (0.96, 1.05)	1.05 (1.01, 1.10)	1.12 (1.07, 1.17)
Model 3	Ref.	1.02 (0.99, 1.05)	1.06 (1.01, 1.11)	1.12 (1.07, 1.17)	1.19 (1.14, 1.25)
Cardiovascular disease-related mortality					
No. events	738	608	670	692	650
Model 1	Ref.	0.93 (0.87, 0.99)	0.92 (0.83, 1.01)	1.01 (0.92, 1.11)	1.15) 1.04, 1.27)
Model 2	Ref.	1.03 (0.96, 1.10)	1.07 (0.97, 1.18)	1.12 (1.02, 1.23)	1.18 (1.06, 1.31)
Model 3	Ref.	1.06 (0.99, 1.13)	1.13 (1.02, 1.25)	1.19 (1.08, 1.30)	1.26 (1.13, 1.40)
Cancer-related mortality					
No. events	1588	1406	1340	1380	1379
Model 1	Ref.	0.89 (0.85, 0.93)	0.84 (0.78, 0.90)	0.88 (0.83, 0.94)	0.95 (0.89, 1.02)
Model 2	Ref.	0.97 (0.93, 1.02)	0.96 (0.90, 1.03)	0.99 (0.93, 1.05)	1.01 (0.94, 1.09)
Model 3	Ref.	0.99 (0.95, 1.04)	1.00 (0.93, 1.07)	1.02 (0.96, 1.09)	1.05 (0.97, 1.13)

Hazard ratios (95% CI) for all-cause, cardiovascular disease-related mortality, and cancer-related mortality during 27 years of follow up, obtained from restricted cubic splines based on Cox proportional hazards models. Model 1 included age and sex; Model 2 included age, sex, BMI, smoking status, smoking packyears, alcohol consumption, education level, physical activity level and living situation; Model 3 adjusted for the covariates in Model 2 plus intakes of wholegrains, refined grains, red meat, processed meat, poultry, dairy, fish, vegetables, fruits, vegetable oils, sugar and confectionary, and soft drinks

Exposure intakes are presented as median [IQR]

Over a 20-year period, individuals with the highest tap water only sourced-nitrate intakes are expected to live, on average, 4 months less [Model 3 RMST_Q5vsQ1_: -4 (-5, -3)] than individuals with the lowest intakes.

#### Time-updated water nitrate intake

When modelling the water nitrate exposure as a time-varying covariate, averaged over a 15-year window, drinking water nitrate concentration was directly associated with all-cause and CVD-related, but not cancer-related mortality (Supplementary Table 2). Compared to participants consuming drinking water with the lowest nitrate concentration (Q1, mean: 0.8 mg/L), those with higher concentrations (Q2 – Q5, mean: 1.4–5.1 mg/L) had a statistically significant 8–14% higher risk of all-cause mortality, after adjusting for demographic, lifestyle and dietary confounders. For CVD-related mortality, statistically significant higher risks between 12 and 16% were seen for participants in quintiles 3–5.

### Stratified analyses

**Fig. 4:**
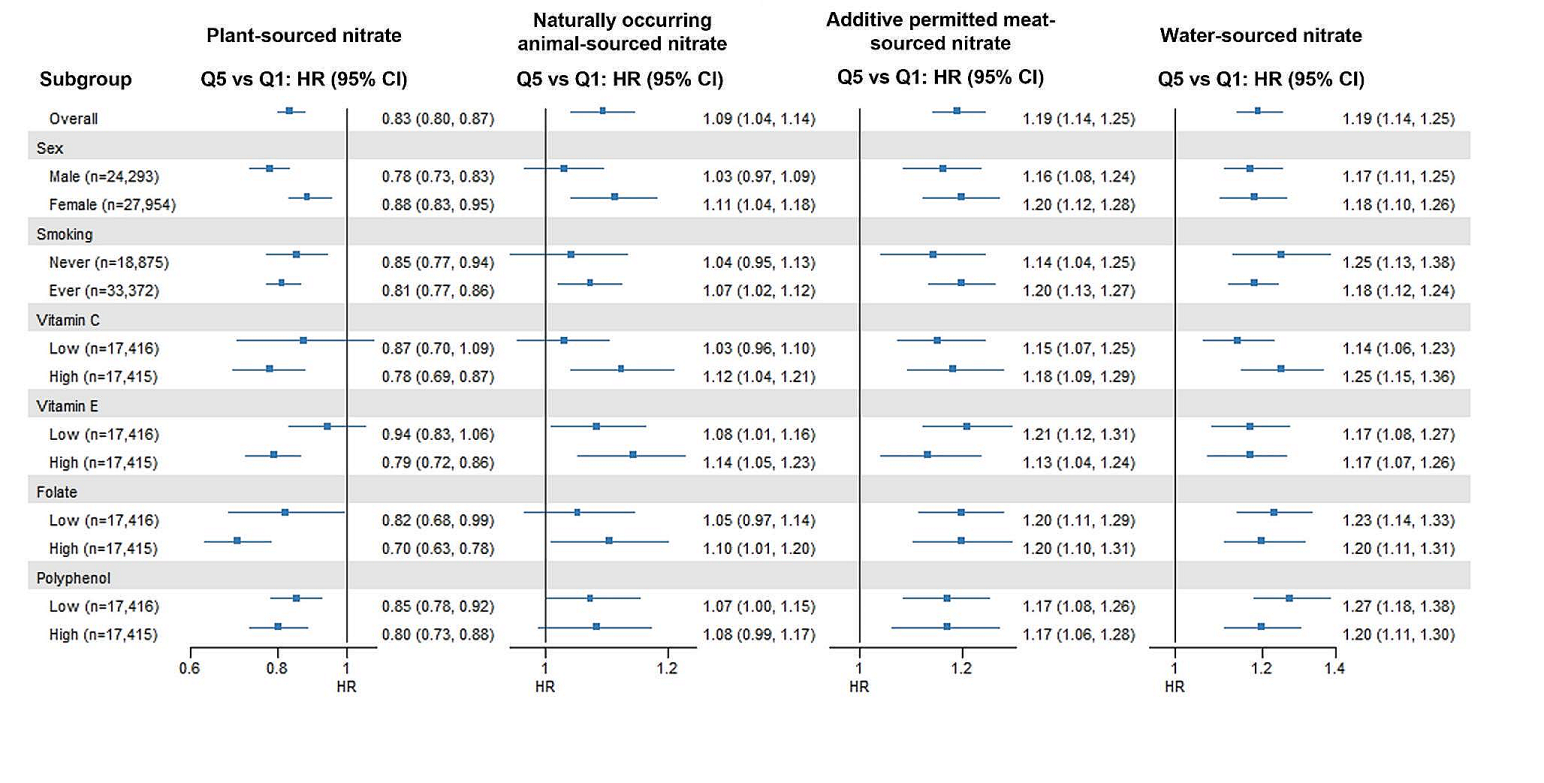
Forest plots depicting associations between plant-sourced, natural occurring animal-sourced, additive permitted meat-sourced and tap water only-sourced nitrate intake and all-cause mortality, stratified by sex, smoking status, and dietary intakes of vitamin C, vitamin E, folate, and polyphenols. Hazard ratios and 95% CIs are derived from Cox proportional hazards models with exposures modelled as restricted cubic splines and are comparing the median intake in quintile 5 to the median intake in quintile 1 (reference). All analyses are adjusted for age, sex, BMI, smoking status, smoking packyears, alcohol consumption, education level, physical activity level, living situation and (i) intakes of red meat, processed meat, poultry, dairy, fish, sugar and confectionary, soft drinks, refined grains, coffee, and tea when the exposure was plant-sourced nitrate, (ii) intakes of wholegrains, refined grains, vegetables, fruits, vegetable oils, sugar and confectionary, soft drinks, refined grains, coffee, and tea when the exposure was animal-sourced nitrate, and (iii) intakes of wholegrains, refined grains, red meat, processed meat, poultry, dairy, fish, vegetables, fruits, vegetable oils, sugar and confectionary, and soft drinks when the exposure was water-sourced nitrate (Model 3)

Evidence of a lower risk of all outcomes is seen for higher intakes of plant-sourced nitrate and nitrite in all subgroups (Fig. 4and Supplementary Figs. 2–6) except that higher intakes of plant-sourced nitrite were not associated with a lower risk of cancer-related mortality in high polyphenol consumers (Supplementary Fig. 6). Evidence of a higher risk of all-cause and cancer-related mortality for higher intakes of additive permitted meat sourced-nitrate and nitrite is seen across all subgroups (Fig. 4 and Supplementary Figs. 2, 4 and 6). Associations for naturally occurring animal sourced-nitrate and all outcomes were weak in the whole analytic cohort; a clear higher risk of CVD-related mortality was only seen in current/former smokers and in participants with high dietary vitamin E intake (Supplementary Fig. 2). Evidence of a higher risk of all outcomes is seen for higher intakes of naturally occurring animal sourced-nitrite in all subgroups (Fig. 4 and Supplementary Figs. 2–6), except for CVD-related mortality in low polyphenol consumers (Supplementary Fig. 5). Evidence of a higher risk of all-cause and CVD-related mortality for higher intakes of tap water-sourced nitrate intake is seen across all subgroups (Fig. 4 and Supplementary Figs. 2 and 3). Tap water nitrate was only associated with a statistically significant higher risk of cancer-related mortality in low polyphenol consumers (Supplementary Fig. 3).

## Discussion

Among 52,247 participants of the Danish Diet Cancer and Health Study, moderate to high intakes of plant-sourced and vegetable-sourced nitrate and nitrite were associated with a 14–24% lower risk of all-cause, CVD-related, and cancer-related mortality. Conversely, higher intakes of naturally occurring animal-sourced nitrate were associated with a 9% and 12% higher risk of all-cause, and CVD-related mortality, respectively, while higher intakes of naturally occurring animal-sourced nitrite were associated with a 25%, 29% and 18% higher risk of all-cause, CVD-related, and cancer-related mortality, respectively. Higher intakes of nitrate and nitrite from additive permitted meat sources were associated with a 12–22% higher risk of all-cause and cancer-related mortality while only additive permitted meat-sourced nitrite was positively associated with CVD-related mortality. Furthermore, participants with a higher intake of tap water-sourced nitrate had a higher risk of all-cause and CVD-related mortality but not cancer-related mortality. No clear patterns emerged when associations were stratified by factors hypothesised to influence the formation of NOCs.

In the present study, higher intakes of plant- and vegetable-sourced nitrate and nitrite were linked to a lower risk of all-cause, CVD-related, and cancer-related mortality. These results build on our prior research which demonstrated that higher intakes of vegetable-sourced nitrate are associated with a lower risk of incident CVD [34], CVD-related mortality [15, 16] and all-cause mortality [16]. While the present study is among the first to explore the relationship between plant-sourced nitrate intake and cancer-related mortality, it is worth noting that no positive association has been reported between intakes of plant-sourced nitrate and incidence (both fatal and non-fatal) of any specific types of cancer [1, 35] while an inverse association between plant-sourced nitrite and gall-bladder cancer has been reported previously [35]. Although the associations in the present study were clear and robust, remaining after additional adjustment for plant-based food intake, we cannot attribute the observed health benefits solely to nitrate/nitrite as their plant-based sources contain many other protective compounds and are themselves associated with a lower risk of CVD, cancer, and mortality [36]. Regardless, our findings underscore the value of promoting higher intakes of nitrate-rich vegetables to mitigate mortality risks while adding to the growing evidence that there is no cause for concern regarding cancer risk from a high consumption of plant-sourced nitrate or nitrite. Indeed, participants in the present study with the highest intakes of plant- and vegetable-sourced nitrate are expected to live, on average, 6 months longer than those with the lowest intakes. Important to acknowledge however is that nitrate and nitrite intakes in this cohort were relatively low [29] and further investigations in populations with higher intakes are necessary to draw firm conclusions around the health consequences of very high intakes of nitrate from plant sources.

In the present study, a higher risk of all-cause and CVD-related mortality was seen for higher intakes of naturally occurring animal-sourced nitrate while a higher risk of all outcomes was seen for higher intakes of additive permitted meat-sourced nitrate and animal-sourced nitrite (both naturally occurring and from additive permitted sources). Notably, associations for naturally occurring animal-sourced nitrate were not robust; they appeared to be strongly influenced by confounding as the association flipped between Model 1 and Model 3 and disappeared after additional adjustment for animal-based food intake. Conversely, associations for naturally occurring animal-sourced nitrite were strong, even though intake was considerably lower than that of nitrate (median intake: 0.9 mg/d vs. 10.7 mg/d for the top quintile), and participants with the highest intakes are expected to live, on average, 4 months less than those with the lowest intakes. Associations for additive permitted meat-sourced nitrate and nitrite were very similar. However, as previously noted [29], the strong correlations between additive-permitted meat-sourced nitrate and nitrite intakes, and processed meat intake (Spearman’s ρ = 0.95), make it challenging to distinguish their separate associations within our dataset, although only associations for additive-permitted meat sourced nitrite remained after additional adjustment for processed meat intake. Our previous research indicates that fresh meats, particularly red meats, might be significant, yet underappreciated, sources of dietary nitrate and nitrite [17]. Indeed, in the Danish Diet Cancer and Health Study, intakes of nitrate and nitrite from meat and fish were roughly 10- and 5-fold higher, respectively, than from meat products where nitrate/nitrite are allowed additives [29]. Factors such as the food matrix, the presence of amines, nitrosation catalysts and inhibitors, and storage conditions can influence the formation of NOCs in foods [1]. While both red and processed meats have the potential to support NOC formation, processed meats have historically raised more concern due to their preservatives and common preparation methods, which are thought to lead to higher NOC formation [37]. However, in the present study, we observed that the higher risk of all-cause, CVD-related, and cancer-related mortality associated with higher intakes of naturally occurring animal-sourced were just as high, if not higher, than those seen for additive-permitted meat-sourced nitrite. This suggests that even naturally occurring nitrite, in the context of animal-based foods, may have adverse health effects. To our knowledge, no studies have investigated the health impacts of animal-sourced nitrate and nitrite on total mortality or CVD, but numerous studies have explored their associations with specific cancers. For instance, higher intakes of nitrate from processed meat have been linked to higher risks of bladder, colorectal, breast, and prostate cancer [1] and higher intakes of nitrite from processed meat have been linked to a higher risk of renal cancer [38], pancreatic cancer [39], stomach cancer [35], and bladder cancer [40]. Contrastingly, higher intakes of nitrite from animal sources have been reported to be associated with a lower risk of small intestine cancer [35], a surprising finding that the authors could not explain and which may be due to chance. Taken together with findings from the present study, there is evidence that nitrate and nitrite from animal sources may increase the risk of cancer. Importantly, the present study shows, for the first time, that higher intakes of nitrite in particular, from animal sources—both inherent and additive—may also increase the risk of all-cause and CVD-related mortality. Indeed, participants with the highest intakes of additive-permitted meat sourced nitrate and nitrite are expected to live, on average, 4 months less than those with the lowest intakes. This may be due to an increase in inflammation and oxidative stress seen with exposure to NOCs [41, 42]. While the literature on the effect of NOCs on cardiovascular health is very sparse, it has been postulated that at high doses nitrosamines may cause cancer but that at low doses, nitrosamines may promote type 2 diabetes mellitus, non-alcoholic steatohepatitis, and Alzheimer’s disease [43]. Thus, the implications of our novel observation are profound, especially since concerns have historically centred around carcinogenesis, and warrant further exploration in other cohorts and preclinical studies.

In the current study, we identified a novel positive association between nitrate intake from drinking water and both all-cause and CVD-related mortality. Upon restricting the exposure to tap water alone (excluding water from beverages like tea and coffee, which contain protective compounds such as polyphenols), the observed associations were even more pronounced. These findings were robust, remaining consistent even after adjusting for dietary factors, excluding participants using private wells, and using a time-updated approach. On an absolute scale, participants with the highest intakes of tap water-sourced nitrate are expected to live, on average, 4 months less than those with the lowest intakes. To date, only one other study, by Houthuijs et al., has explored the relationship between water nitrate and all-cause mortality [44]. Their nationwide study, aiming at evaluating drinking water safety in the Netherlands, reported no association between water nitrate and non-accidental mortality; although the risk did appear to be significantly higher for some intake groups, the pattern was not consistent. It is worth noting that their reference group was water nitrate concentrations of 5 mg/L or less. In contrast, our study demonstrated an 8% higher risk of mortality at water nitrate concentrations of 1.4 mg/L, with the association plateauing at a risk of approximately 13–14% at concentrations around 2.4 mg/L. It is possible that the choice of reference group in Houthuijs’ study might have obscured potential associations. That we found an association with all-cause and CVD-related but not cancer-related mortality was surprising as concerns around the safety of drinking water nitrate, besides methemoglobinemia and birth defects, have historically centered around cancer [45]. However, our findings do align with the hypothesis that at low concentrations, such as those seen in this cohort, nitrosamines may promote other diseases, particular those mediated by insulin resistance, while at high concentrations, they may be carcinogenic [43]. With evidence available to date, it is unclear why nitrate from water, especially at the low concentrations observed in the present study, was associated with a higher risk of CVD-related mortality. In opposition to our findings, in 15,549 participants of EPIC-Norfolk cohort, water nitrate concentration was not associated with CVD risk, however, the authors report interaction between water nitrate and water sulfate concentrations such that water nitrate was only associated with a lower blood pressure at low sulfate concentrations and at high sulfate concentrations, the association reversed and nitrate was associated with higher blood pressure [46]. Mechanisms postulated by the authors include cross-talk between sulfur and nitrogen metabolic pathways, competition at the level of the microbiome in either the gut or the oral cavity, and/or modulation of the gut microbiome. Unfortunately, our study lacks data on water sulfate concentrations, but such findings underscore the need for further exploration in different cohorts. For over four decades, nitrate-contaminated drinking water and its potential association with cancer have raised public health concerns [47]. While we found no association between drinking water nitrate intake and cancer-related mortality, it is essential to recognise the vast differences in the aetiologies of various cancers. Therefore, future research should investigate the links between nitrate intake and specific cancer types. Recent research demonstrating that reducing the nitrate concentration of drinking water in Denmark to below 4 mg/L could save around $302 million per year by reducing healthcare costs related to colorectal cancer [48] underscores the critical importance of this study’s findings which indicate that the detrimental health consequences of nitrate in drinking water likely extend beyond colorectal cancer and can occur at even lower nitrate concentrations. Hopefully more research like this will spur efforts to reduce the nitrate concentration of drinking water as, given the time-lag, even if nitrogen leaching from agriculture in vulnerable catchments is limited, the effects in groundwater sources of drinking water may not be seen until decades later [49]. Therefore, other mitigation measures to reduce nitrate levels in drinking water such as water supply infrastructural changes or water treatment may be necessary to consider [48].

Beyond the intrinsic factors of the food matrix containing nitrate/nitrite, external elements such as smoking habits and the broader dietary pattern are believed to influence their metabolic pathways in the body. Smoking is hypothesised to elevate the formation of NOCs as smokers have a higher concentration of thiocyanate—a strong catalyst of the nitrosation of amines and a competitor for salivary gland uptake—in their saliva than non-smokers [50]. Conversely, the concurrent ingestion of vitamin C, vitamin E, folate, and polyphenols are thought to inhibit nitrosation by scavenging nitrosating agents [51]. In the present study, no clear patterns in our stratified analyses emerged to strongly support this hypothesis. However, the timing of consumption of these protective compounds may be critical, a factor that we cannot account for in the present study; clinical trials may be more suitable to conclusively address this issue.

This observational study brings with it inherent limitations that preclude establishing causality. Although we controlled for known confounders to the best of our ability, residual confounding—namely by sociodemographic, dietary, water pollutant, and air pollutant confounders—cannot be ruled out. Though sourced from comprehensive databases, the nitrate and nitrite intake estimates were based on an FFQ, and we likely missed the contribution of less common high-nitrate foods and factors like cultivation practices and storage conditions which can influence nitrate concentrations in foods. The study’s inability to distinguish the effects of nitrate/nitrite from other food components, potential confounder changes after baseline, and the possibility of misclassification bias in the Danish Register of Causes of Death [22], necessitate a cautious interpretation. Furthermore, the concentration range for drinking water nitrate was low and not representative of the entire population of Denmark where approximately 10% of the population are exposed to levels exceeding 9 mg/L [48], we did not have information on drinking water nitrite concentration, nor the drinking water nitrate concentration at the participants’ place of work, and drinking water nitrate may be a proxy for other agricultural pollutants. Additionally, the largely Caucasian Danish cohort may limit the broader applicability of our findings. However, the study’s strengths are notable. We tracked a large adult cohort over 27 years with minimal loss to follow-up, leveraging comprehensive databases for food and beverage nitrate content. All observed associations, except for nitrate from animal-sourced foods, were robust and remained after additional adjustment for the food group source of the dietary exposure in sensitivity analyses. The integration of water intake with longitudinal drinking water nitrate levels, specific to place of residency, offers a unique and more comprehensive view of water nitrate exposure than has been seen previously. This approach enhanced the depth of our analysis, providing a clearer picture of both baseline and time-updated drinking water-sourced nitrate intake, thus strengthening confidence in observed associations.

## Conclusion

Within the Danish Diet, Cancer and Health Study, we report a noteworthy dichotomy in the associations between nitrate and nitrite intake sources and mortality risks. Plant and vegetable-sourced nitrate and nitrite intakes were inversely associated with mortality. Conversely, intakes of animal-sourced nitrate and nitrite, whether naturally occurring or additive, were associated with a higher risk of mortality. Notably, our study provides novel evidence that higher intakes of nitrate and nitrite from animal sources, and nitrate from drinking water even at low concentrations, may be linked to a higher risk of all-cause and CVD-related mortality. These findings challenge historical concerns primarily focused on cancer and call for further research to be done to fully elucidate the health consequences of source-dependent dietary nitrate and nitrite intakes.

## Electronic supplementary material

Below is the link to the electronic supplementary material.

Supplementary Material 1
