# Supplementary material for: Source-specific nitrate intake and all-cause mortality in the Danish Diet, Cancer, and Health Study
Source: Eur J Epidemiol. 2024 May 28;39(8):925–42. doi: 10.1007/s10654-024-01133-5 (PMC11410901; doi:10.1007/s10654-024-01133-5)
Supplement: Supplementary file 1 — Supplementary Material 1 [file 10654_2024_1133_MOESM1_ESM.docx]

**Source-specific nitrate intake and all-cause mortality in the Danish Diet, Cancer, and Health Study**

*Bondonno et al.,*

**Supplementary Material**

| **Supplementary Table 1.** Definitions for determining prevalent comorbidities at baseline | |
| --- | --- |
| **Prevalent disease** | **Definition** |
| Ischemic heart disease | ICD-8 diagnosis [410-414] or ICD-10 diagnosis [I20-I25] in the Danish National Patient Register prior to baseline |
| Ischemic stroke | ICD-8 diagnosis [433-434] or ICD-10 diagnosis [I63] in the Danish National Patient Register prior to baseline |
| Peripheral artery disease | ICD-8 diagnosis [440-444] or ICD-10 diagnosis [I70-I74] in the Danish National Patient Register prior to baseline |
| Heart failure | ICD-8 diagnosis [4270-4271] or ICD-10 diagnosis [I42, I50, I110, J81] in the Danish National Patient Register prior to baseline |
| Atrial fibrillation | ICD-8 diagnosis [42793-42794] or ICD-10 diagnosis [I48] in the Danish National Patient Register prior to baseline |
| Chronic obstructive pulmonary disease | ICD-8 diagnosis [491-493] or ICD-10 diagnosis [J42-J44] in the Danish National Patient Register or a record of COPD in the RUKS registry^1^ prior to baseline |
| Diabetes | A record of either type 1 or type 2 diabetes in the RUKS registry^1^ prior to baseline |
| Chronic kidney disease | ICD-8 diagnosis [580-584] or ICD-10 diagnosis [N02-N08, N11-N12, N14, N18-N19, N26, N158-N160, N162-N164, N168, Q61, E102, E112, E132, E142, I120, M321B] in the Danish National Patient Register or a record of COPD in the RUKS registry^1^ prior to baseline |

ICD; International Classification of Diseases [the 8^th^ revision (ICD-8) until 1993 and the 10^th^ revision (ICD-10) from 1994 to present]. ^1^ Algorithms described in more detail here (1)

Diet, Cancer, and Health Study participants recruited (n=57,053)

Ineligible participants (n=590)

Subsequent cancer diagnosis attributed to diagnostic criteria present before participation, emigration and consent withdrawn

Total cohort participants eligible for this study (n=56,463)

Excluded participants (n=4216)

- Prevalent cardiovascular disease [n=2705]

Missing data:

- Food nitrate/nitrite [n=48]
- Water nitrate [n=400]
- Other covariates [n=1063]

Total participants included in the current study **(n=52,247)**

**Supplementary Figure 1:** CONSORT flow diagram of the DCH study participants included in present study

| **Supplementary Table 2. Hazards ratio of mortality subtypes by quintiles of drinking water nitrate concentration from time-updated analyses** | | | | | |
| --- | --- | --- | --- | --- | --- |
|  | Q1 | Q2 | Q3 | Q4 | Q5 |
| Concentration* (mg/L) | 0.83 (0.71 – 1.00) | 1.38 (1.36 – 1.44) | 1.78 (1.76 – 1.96) | 2.37 (2.29 – 2.88) | 5.09 (4.20 – 7.13) |
| All-cause mortality | |  |  |  |  |
| No. events | 1663 | 2065 | 1919 | 2196 | 1959 |
| Model 1 | Ref. | 1.11 (1.07, 1.16) | 1.17 (1.11, 1.23) | 1.22 (1.16, 1.28) | 1.26 (1.18, 1.34) |
| Model 2 | Ref. | 1.08 (1.04, 1.13) | 1.11 (1.06, 1.17) | 1.14 (1.08, 1.19) | 1.14 (1.07, 1.22) |
| Model 3 | Ref. | 1.08 (1.04, 1.13) | 1.11 (1.06, 1.17) | 1.13 (1.08, 1.19) | 1.14 (1.07, 1.21) |
| Cardiovascular disease-related mortality | | |  |  |  |
| No. events | 312 | 419 | 366 | 428 | 408 |
| Model 1 | Ref. | 1.11 (1.01, 1.22) | 1.18 (1.05, 1.31) | 1.25 (1.12, 1.40) | 1.30 (1.12, 1.50) |
| Model 2 | Ref. | 1.09 (0.99, 1.20) | 1.13 (1.01, 1.27) | 1.17 (1.05, 1.31) | 1.16 (1.01, 1.34) |
| Model 3 | Ref. | 1.08 (0.98, 1.19) | 1.12 (1.00, 1.26) | 1.17 (1.04, 1.30) | 1.16 (1.00, 1.34) |
| Cancer-related mortality | | |  |  |  |
| No. events | 835 | 965 | 907 | 1020 | 879 |
| Model 1 | Ref. | 1.07 (1.00, 1.13) | 1.10 (1.02, 1.17) | 1.12 (1.05, 1.20) | 1.15 (1.05, 1.26) |
| Model 2 | Ref. | 1.04 (0.98, 1.10) | 1.05 (0.98, 1.12) | 1.05 (0.98, 1.13) | 1.06 (0.96, 1.16) |
| Model 3 | Ref. | 1.04 (0.98, 1.11) | 1.05 (0.98, 1.13) | 1.06 (0.99, 1.13) | 1.06 (0.96, 1.16) |
| Hazard ratios (95% CI) for all-cause, cardiovascular disease-related mortality, and cancer-related mortality during 23 years of follow up, obtained from restricted cubic splines based on Cox proportional hazards models with age as the underlying timescale. Model 1 included sex and time since study entrance; Model 2 included sex, time since study entrance, BMI, smoking status, smoking packyears, alcohol consumption, education level, physical activity level, living situation and baseline tap water intake; Model 3 adjusted for the covariates in Model 2 plus intakes of wholegrains, refined grains, red meat, processed meat, poultry, dairy, fish, vegetables, fruits, vegetable oils, sugar and confectionary, soft drinks.  *Baseline 15-year average concentration of drinking water nitrate presented as median (IQR) | | | | | |


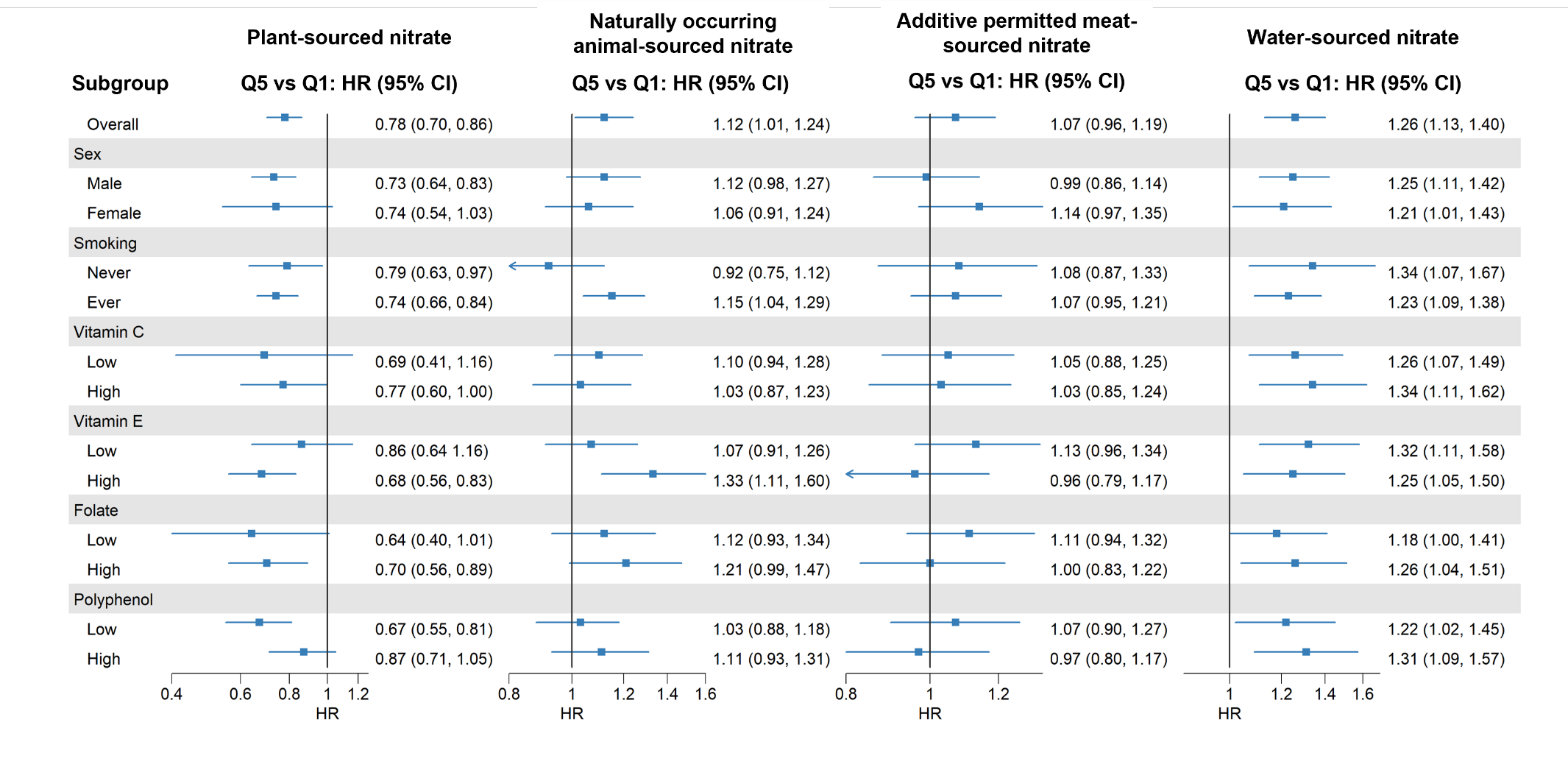


**Supplementary Figure 2.** Forest plots depicting associations between plant-sourced, natural occurring animal-sourced, additive permitted meat-sourced and tap water only-sourced nitrate intake and cardiovascular disease-related mortality, stratified by sex, smoking status, and dietary intakes of vitamin C, vitamin E, folate, and polyphenols. Hazard ratios and 95% CIs are derived from Cox proportional hazards models with exposures modelled as restricted cubic splines and are comparing the median intake in quintile 5 to the median intake in quintile 1 (reference). All analyses are adjusted for age, sex, BMI, smoking status, smoking packyears, alcohol consumption, education level, physical activity level, living situation and i) intakes of red meat, processed meat, poultry, dairy, fish, sugar and confectionary, soft drinks, refined grains, coffee, and tea when the exposure was plant-sourced nitrate, ii) intakes of wholegrains, refined grains, vegetables, fruits, vegetable oils, sugar and confectionary, soft drinks, refined grains, coffee, and tea when the exposure was animal-sourced nitrate, and iii) intakes of wholegrains, refined grains, red meat, processed meat, poultry, dairy, fish, vegetables, fruits, vegetable oils, sugar and confectionary, and soft drinks when the exposure was water-sourced nitrate (Model 3).


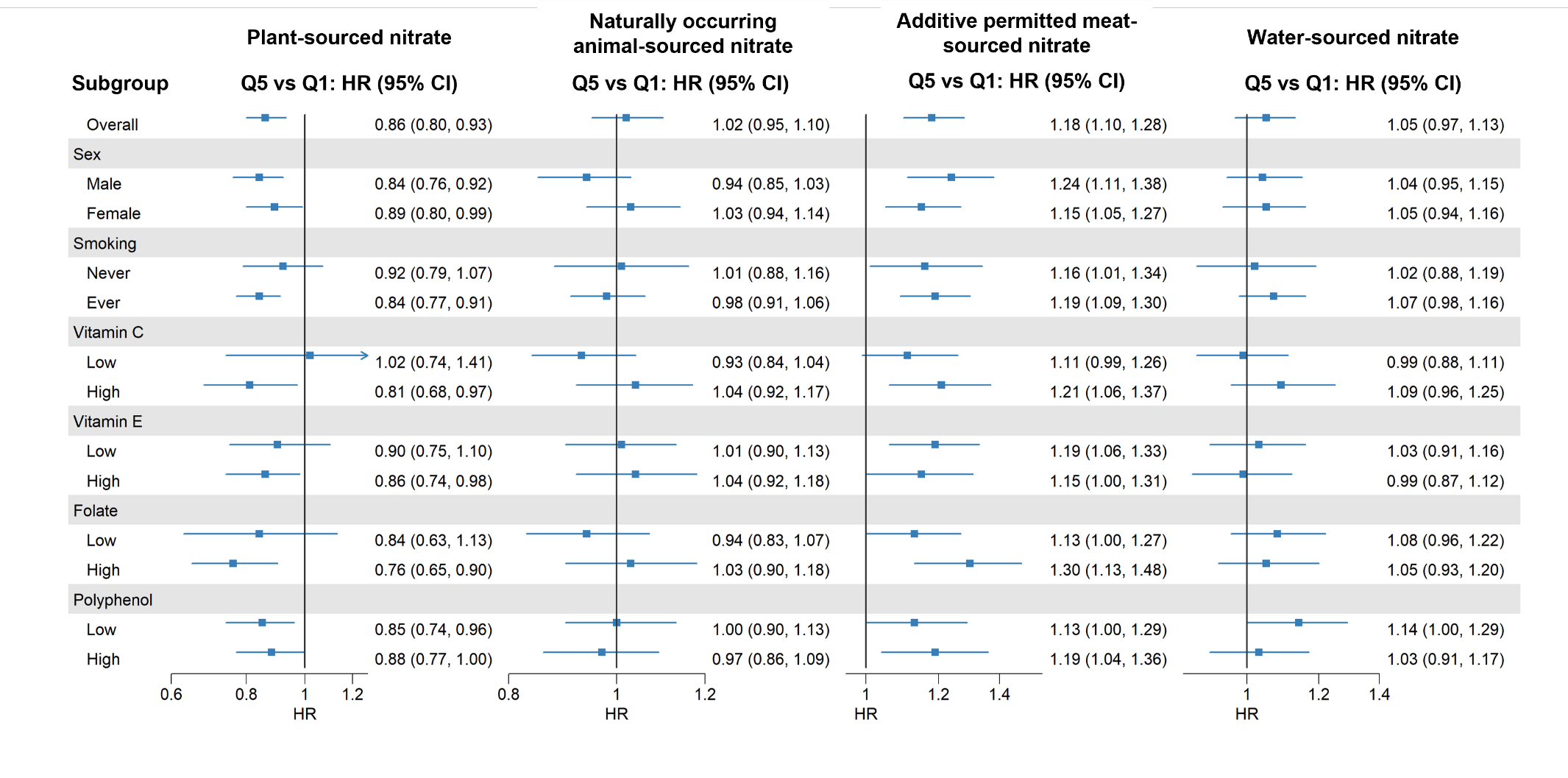


**Supplementary Figure 3.** Forest plots depicting associations between plant-sourced, natural occurring animal-sourced, additive permitted meat-sourced and tap water only-sourced nitrate intake and cancer-related mortality, stratified by sex, smoking status, and dietary intakes of vitamin C, vitamin E, folate, and polyphenols. Hazard ratios and 95% CIs are derived from Cox proportional hazards models with exposures modelled as restricted cubic splines and are comparing the median intake in quintile 5 to the median intake in quintile 1 (reference). All analyses are adjusted for age, sex, BMI, smoking status, smoking packyears, alcohol consumption, education level, physical activity level, living situation and i) intakes of red meat, processed meat, poultry, dairy, fish, sugar and confectionary, soft drinks, refined grains, coffee, and tea when the exposure was plant-sourced nitrate, ii) intakes of wholegrains, refined grains, vegetables, fruits, vegetable oils, sugar and confectionary, soft drinks, refined grains, coffee, and tea when the exposure was animal-sourced nitrate, and iii) intakes of wholegrains, refined grains, red meat, processed meat, poultry, dairy, fish, vegetables, fruits, vegetable oils, sugar and confectionary, and soft drinks when the exposure was water-sourced nitrate (Model 3).


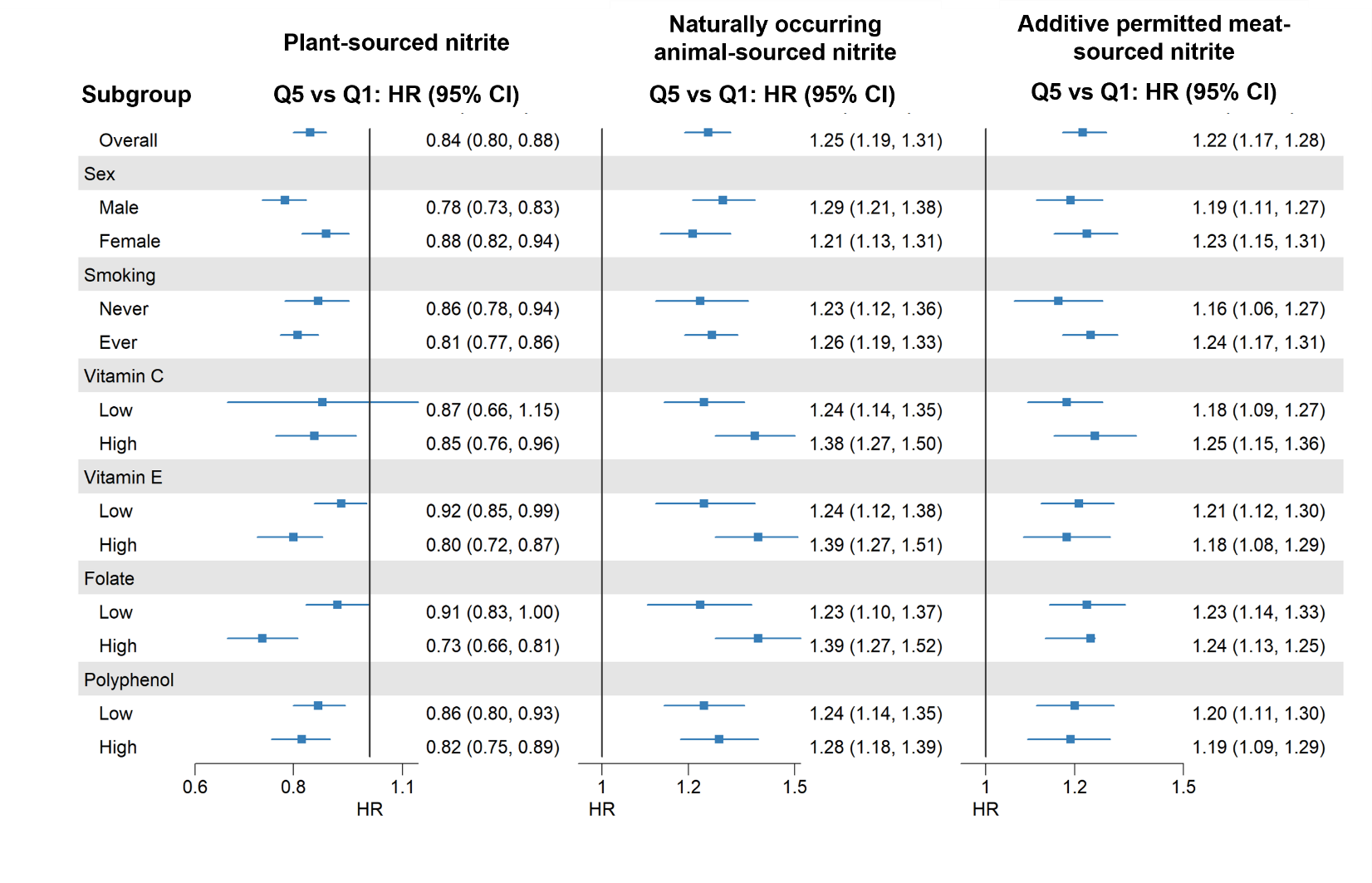
**Supplementary Figure 4.** Forest plots depicting associations between plant-sourced, natural occurring animal-sourced and additive permitted meat-sourced nitrite intake and all-cause mortality, stratified by sex, smoking status, and dietary intakes of vitamin C, vitamin E, folate, and polyphenols. Hazard ratios and 95% CIs are derived from Cox proportional hazards models with exposures modelled as restricted cubic splines and are comparing the median intake in quintile 5 to the median intake in quintile 1 (reference). All analyses are adjusted for age, sex, BMI, smoking status, smoking packyears, alcohol consumption, education level, physical activity level, living situation and i) intakes of red meat, processed meat, poultry, dairy, fish, sugar and confectionary, soft drinks, refined grains, coffee, and tea when the exposure was plant-sourced nitrate, and ii) intakes of wholegrains, refined grains, vegetables, fruits, vegetable oils, sugar and confectionary, soft drinks, refined grains, coffee, and tea when the exposure was animal-sourced nitrate (Model 3).

**
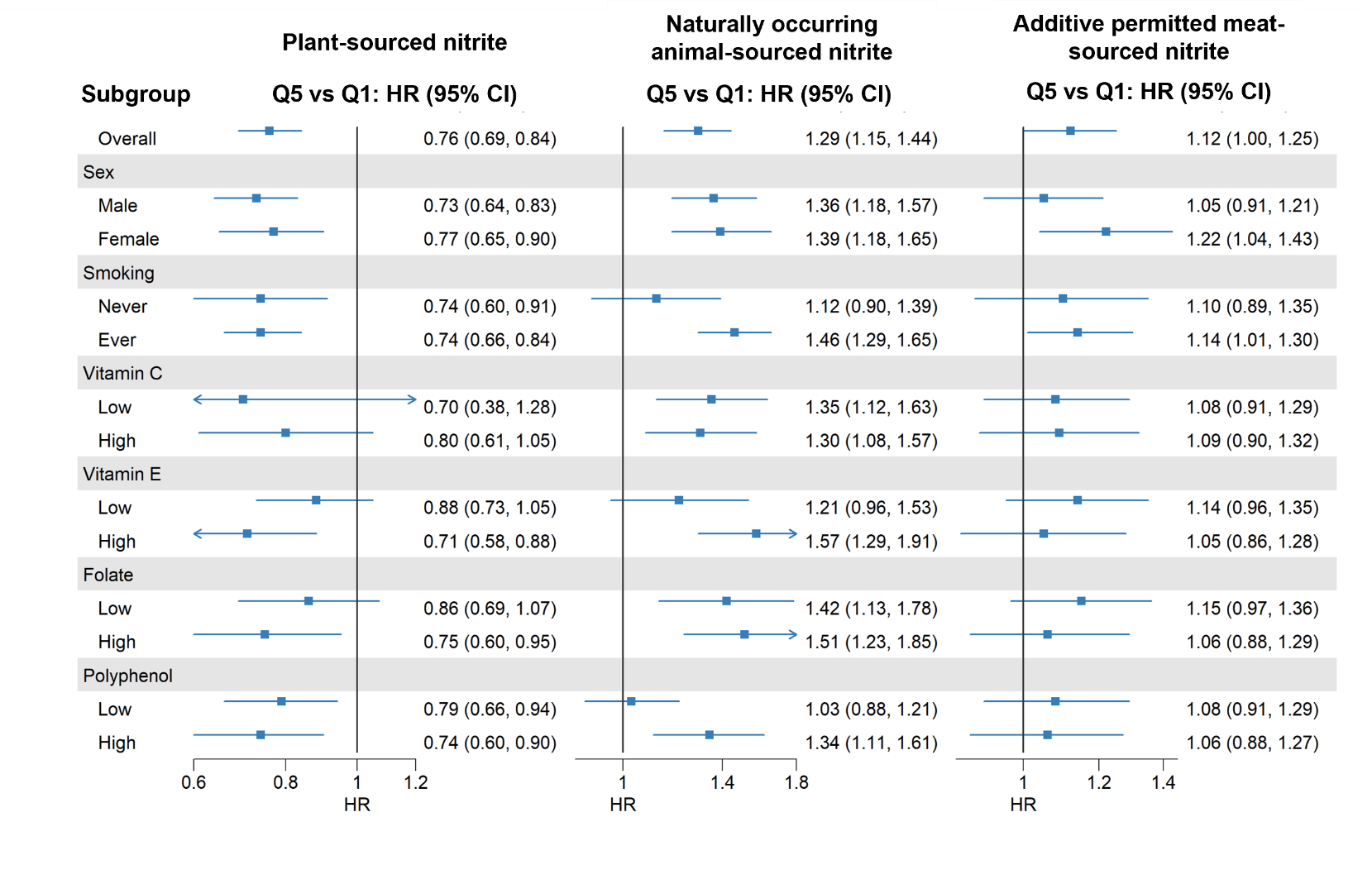
Supplementary Figure 5.** Forest plots depicting associations between plant-sourced, natural occurring animal-sourced and additive permitted meat-sourced nitrite intake and cardiovascular disease-related mortality, stratified by sex, smoking status, and dietary intakes of vitamin C, vitamin E, folate, and polyphenols. Hazard ratios and 95% CIs are derived from Cox proportional hazards models with exposures modelled as restricted cubic splines and are comparing the median intake in quintile 5 to the median intake in quintile 1 (reference). All analyses are adjusted for age, sex, BMI, smoking status, smoking packyears, alcohol consumption, education level, physical activity level, living situation and i) intakes of red meat, processed meat, poultry, dairy, fish, sugar and confectionary, soft drinks, refined grains, coffee, and tea when the exposure was plant-sourced nitrate, and ii) intakes of wholegrains, refined grains, vegetables, fruits, vegetable oils, sugar and confectionary, soft drinks, refined grains, coffee, and tea when the exposure was animal-sourced nitrate (Model 3).

**
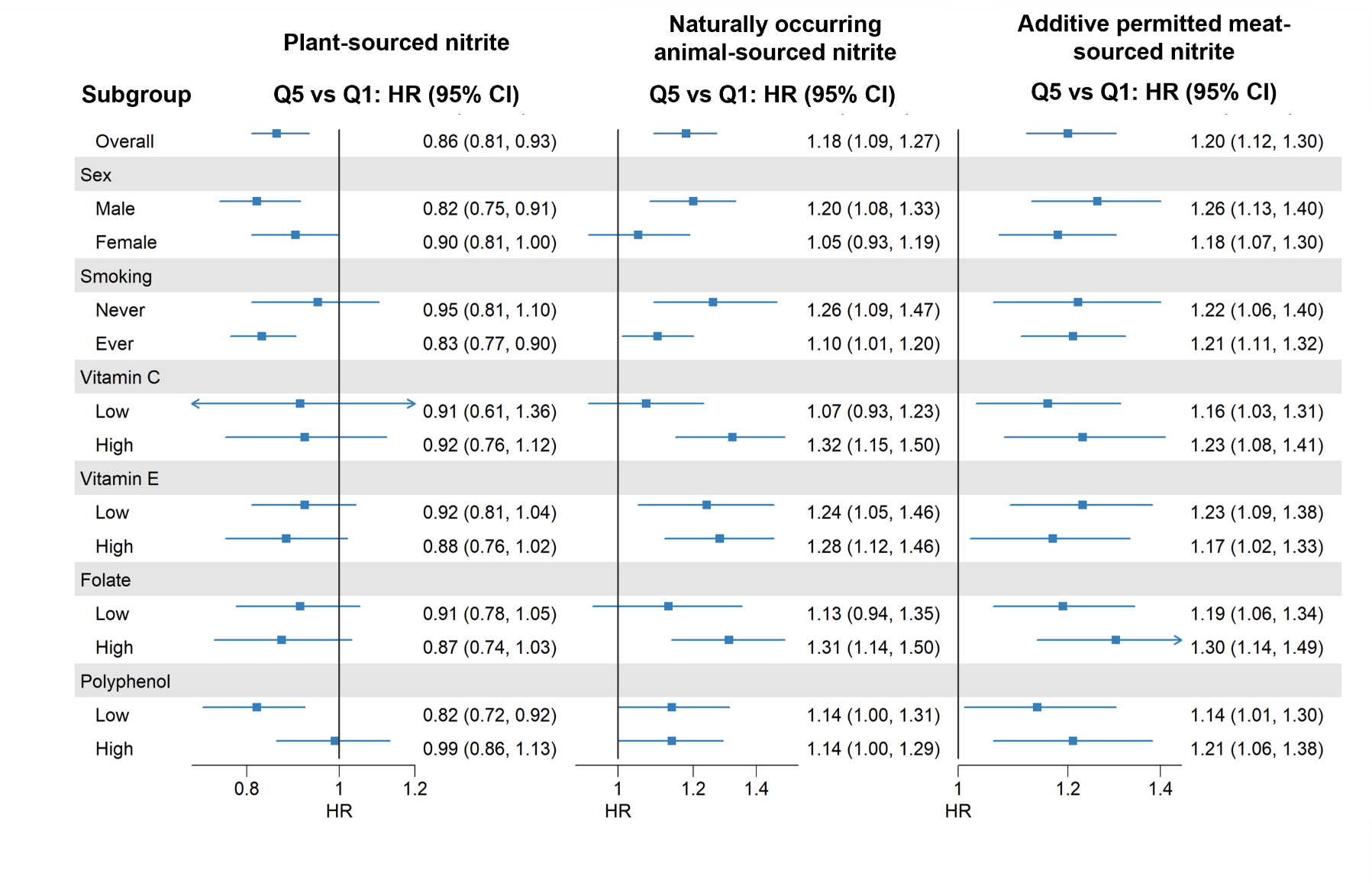
Supplementary Figure 6.** Forest plots depicting associations between plant-sourced, natural occurring animal-sourced and additive permitted meat-sourced nitrite intake and cancer-related mortality, stratified by sex, smoking status, and dietary intakes of vitamin C, vitamin E, folate, and polyphenols. Hazard ratios and 95% CIs are derived from Cox proportional hazards models with exposures modelled as restricted cubic splines and are comparing the median intake in quintile 5 to the median intake in quintile 1 (reference). All analyses are adjusted for age, sex, BMI, smoking status, smoking packyears, alcohol consumption, education level, physical activity level, living situation and i) intakes of red meat, processed meat, poultry, dairy, fish, sugar and confectionary, soft drinks, refined grains, coffee, and tea when the exposure was plant-sourced nitrate, and ii) intakes of wholegrains, refined grains, vegetables, fruits, vegetable oils, sugar and confectionary, soft drinks, refined grains, coffee, and tea when the exposure was animal-sourced nitrate (Model 3).

**References**

1. Sundhedsdatastyrelsen [Danish Board of Health Data], Algoritmer for udvalgte kroniske sygdomme og svære psykiske lidelser [Algorithms for selected chronic diseases and severe psychiatric conditions]. <https://www.esundhed.dk/Dokumentation/-/media/Files/Publikationer/Emner/Operationer-og-diagnoser/Udvalgte-kroniske-sygdomme-svaere-psykiske-lidelser/RUKS-Algoritmer-2021.ashx?la=da&hash=34DEC5295DF1442A92AD711C43EDD7C2> Accessed 21 Apr 2023.
